# Interleukin-27 Promotes Divergent Effects on HIV-1 Infection in Peripheral Blood Mononuclear Cells through BST-2/Tetherin

**DOI:** 10.1128/jvi.01752-22

**Published:** 2023-01-05

**Authors:** Jairo R. Temerozo, Pedro L. C. Ferreira, Leandra Linhares-Lacerda, Rhaíssa C. Vieira, Bruno Cister-Alves, Livia Gobbo, Marcelo Ribeiro-Alves, Rubem F. S. Menna-Barreto, Dumith Chequer Bou-Habib

**Affiliations:** a Laboratory on Thymus Research, Oswaldo Cruz Institute/Fiocruz, Rio de Janeiro, Brazil; b Laboratory of Immunobiology of Leishmaniasis, Department of Immunology, Paulo de Goes Institute of Microbiology, Federal University of Rio de Janeiro, Rio de Janeiro, Brazil; c Laboratory of Cellular Biology, Oswaldo Cruz Institute/Fiocruz, Rio de Janeiro, Brazil; d HIV/AIDS Clinical Research Center, Evandro Chagas National Institute of Infectology, Fiocruz, Rio de Janeiro, Brazil; e National Institute of Science and Technology on Neuroimmunomodulation, Rio de Janeiro, Brazil; Icahn School of Medicine at Mount Sinai

**Keywords:** HIV-1, IL-27, BST-2/Tetherin, PBMCs

## Abstract

Interleukin-27 (IL-27) is able to inhibit HIV-1 replication in peripheral blood mononuclear cells (PBMCs), macrophages, and dendritic cells. Here, we identify that IL-27 can produce opposing effects on HIV-1 replication in PBMCs and that the HIV-1 restriction factor BST-2/Tetherin is involved in both inhibitory and enhancing effects on HIV-1 infection induced by IL-27. IL-27 inhibited HIV-1 replication when added to cells 2 h after infection, promoting the prototypical BST-2/Tetherin-induced virion accumulation at the cell membrane of HIV-1-infected PBMCs. BST-2/Tetherin gene expression was significantly upregulated in the IL-27-treated PBMCs, with a simultaneous increase in the number of BST-2/Tetherin^+^ cells. The silencing of BST-2/Tetherin diminished the anti-HIV-1 effect of IL-27. In contrast, IL-27 increased HIV-1 production when added to infected cells 4 days after infection. This enhancing effect was prevented by BST-2/Tetherin gene knockdown, which also permitted IL-27 to function again as an HIV-1 inhibitory factor. These contrasting roles of IL-27 were associated with the dynamic of viral production, since the IL-27-mediated enhancement of virus replication was prevented by antiretroviral treatment of infected cells, as well as by keeping cells under agitation to avoid cell-to-cell contact. Likewise, inhibition of CD11a, an integrin associated with HIV-1 cell-to-cell transmission, abrogated the IL-27 enhancement of HIV-1 production. Our findings illustrate the complexity of the HIV-1–host interactions and may impact the potential therapeutic use of IL-27 and other soluble mediators that induce BST-2/Tetherin expression for HIV-1 infection.

**IMPORTANCE** Here, we describe new findings related to the ability of the cytokine IL-27 to regulate the growth of HIV-1 in CD4^+^ T lymphocytes. IL-27 has long been considered a potent inhibitor of HIV-1 replication, a notion based on several reports showing that this cytokine controls HIV-1 infection in peripheral blood mononuclear cells (PBMCs), monocyte-derived macrophages, and dendritic cells. However, our present results are contrary to the current knowledge that IL-27 acts only as a powerful downregulator of HIV-1 replication. We observed that IL-27 can either prevent or enhance viral growth in PBMCs, an outcome dependent on when this cytokine is added to the infected cells. We detected that the increase of HIV-1 dissemination is due to enhanced cell-to-cell transmission with the involvement of the interferon-induced HIV-1 restriction factor BST-2/Tetherin and CD11a (LFA-1), an integrin that participates in formation of virological synapse.

## INTRODUCTION

Interleukin-27 (IL-27), a member of the cytokine family of IL-6, IL-12, and IL-23, is produced by antigen-presenting cells during the initial phase of the immune response and plays critical regulatory roles in the functioning of the immune system (1). IL-27 is a heterodimer comprising the protein subunits p28 and EBI3 (Epstein-Barr virus-induced gene 3) (1, 2), and its receptor is a heterodimer formed from the cytokine type 1 common receptor WSX-1 and gp130, which is shared with the IL-6 receptor. Many cells, such as monocytes, macrophages, dendritic cells, T and B lymphocytes, and NK and mast cells, express the IL-27 receptor (3). IL-27 presents a variety of regulatory functions, such as the development of the Th1 and Th2 immune responses through inducing gamma interferon (IFN-γ) production by T cells (1) and inhibiting the expression of the transcription factor GATA-3 (4), production of pro- and anti-inflammatory mediators by activating STAT and NF-κB transcription factors (5–7), and priming naive T cells for IL-12 action, thus favoring cell polarization toward the Th1 phenotype (1).

Infection with human immunodeficiency virus type 1 (HIV-1), the etiological agent of AIDS, causes a substantial loss of CD4^+^ T cells in gastrointestinal-associated lymphoid tissues (8) and leads to chronic immune activation (9) and a proinflammatory condition in the sites of viral growth (10), thus favoring HIV-1 propagation due to the influx of new target cells. The increased expression of several inflammatory cytokines able to regulate HIV-1 replication in the lymphoid tissues of HIV-1-infected patients (11) likely also facilitates HIV-1 dissemination. It is possible that HIV-1 propagation in the lymphoid tissues occurs mainly through cell-to-cell transmission, with dendritic cells and macrophages playing relevant roles in the process of viral particle transmission to CD4^+^ T lymphocytes (12, 13).

Works reporting whether HIV-1 infection impacts circulating levels of IL-27 are rare and found conflicting results. While He et al. (14) showed that IL-27 titer was significantly upregulated in HIV-1-infected individuals and that HIV-1 viral load negatively correlated with IL-27 titers, Swaminathan et al. (15) reported that IL-27 levels were not significantly altered in patients with HIV-1. On the other hand, several *in vitro* studies have shown that IL-27 reduces HIV-1 infection in all types of HIV-1-target cells, such as peripheral blood mononuclear cells (PBMCs), CD4^+^ T cells, monocyte-derived macrophages, and dendritic cells (16–20). It has also been shown that IL-27 generates an anti-HIV-1 setting by activating a number of interferon (IFN)-stimulated genes (ISGs), including MX1, OAS, PKR, APOBEC3G (20), and BST-2/Tetherin (21), in human macrophages, monocytes, and T cells in an IFN-independent manner. Accordingly, the IL-27-induced inhibition of HIV-1 replication can occur by either type 1 IFN-dependent (19) or -independent (17, 20) pathways.

The BST-2/Tetherin protein is an IFN-induced virus restriction factor that prevents the release of HIV-1 particles by anchoring the budding virions to the membrane of infected cells (22–24). Although it is believed that the BST-2/Tetherin ability to impede virus release from the cell membrane, thus restricting HIV-1 cell-free propagation, contributes for a lower virus burden, studies have shown that the BST-2/Tetherin-mediated virion accumulation at the cell surface may actually increase viral production by favoring HIV-1 cell-to-cell dissemination (25). Given that the expression of BST-2/Tetherin may be regulated by IL-27, investigations are needed to elucidate whether this cytokine may also present contradictory roles during HIV-1 infection. Here, we describe novel findings divergent of the current knowledge that IL-27 acts solely as an inhibitor of HIV-1 replication, since we observed that it is capable of either preventing or enhancing viral growth in PBMCs and that the BST-2/Tetherin protein is implicated in both outcomes.

## RESULTS

### IL-27 increases the expression of BST-2/Tetherin and promotes virion accumulation at the cell membrane of HIV-1-infected PBMCs.

**FIG 1:**
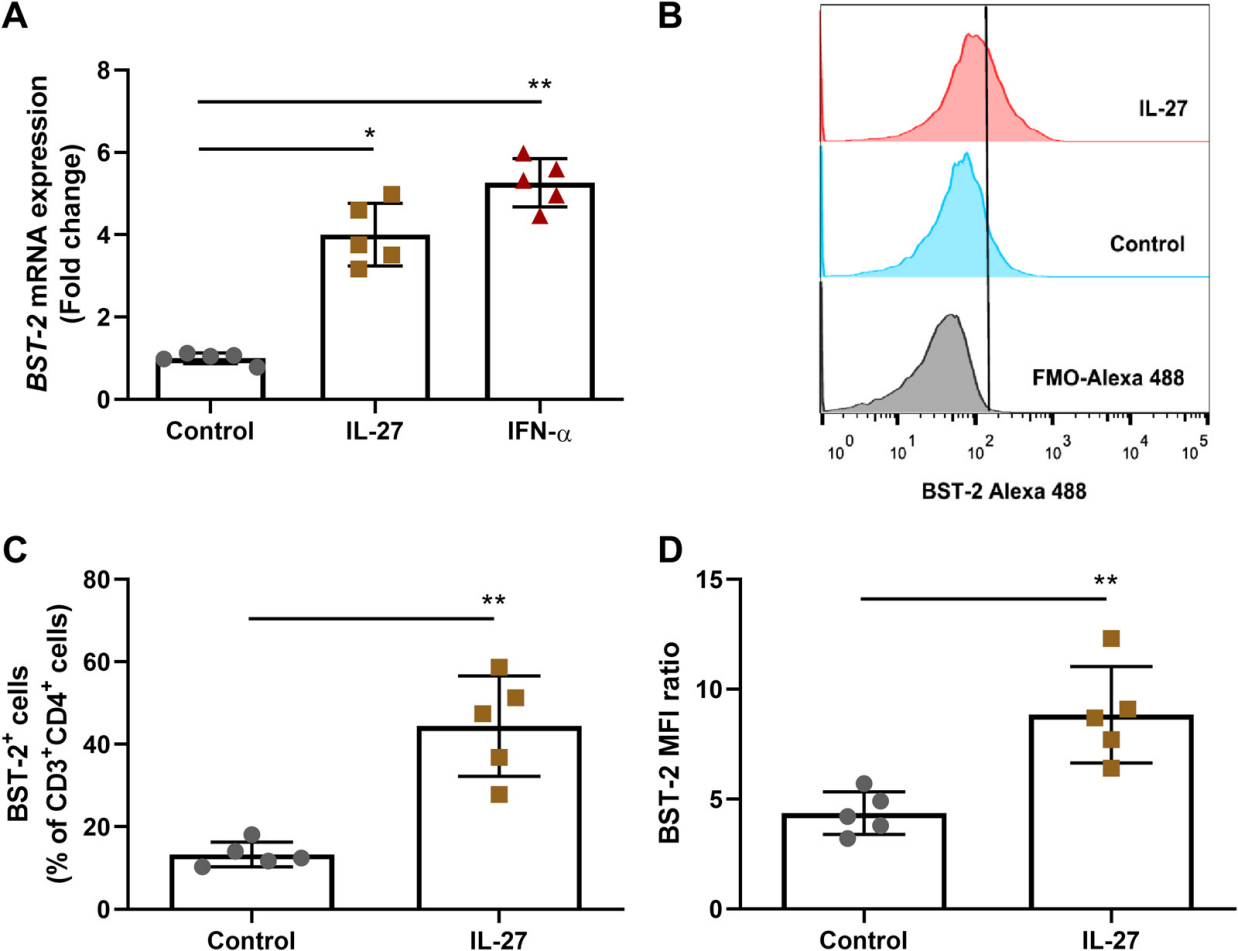
IL-27 promotes the expression of BST-2/Tetherin in PBMCs. (A) PBMCs were exposed to IL-27 (100 ng/mL) or IFN-α (10 ng/mL) and, after 18 h, the expression of *BST2* (Tetherin) was analyzed by real-time PCR. (B to D) HIV-1-infected PBMCs were treated with IL-27 (100 ng/mL) and, after 24 h, BST-2/Tetherin expression in cells within the CD3^+^ CD4^+^ gate was analyzed by flow cytometry. (B) Representative histogram of BST-2/Tetherin expression in the CD3^+^ CD4^+^ populations. (C) Percentage of BST-2/Tetherin1 cells in the CD3^+^ CD4^+^ population. (D) Median fluorescence intensity (MFI) of BST-2/Tetherin in positive cells, calculated as the MFI ratio = specific MFI/FMO MFI. *, *P* < 0.05; **, *P* < 0.01 (one-way ANOVA with Tukey’s multiple-comparison test, *n* = 5 [A], and Student *t* test, *n* = 5 [B to D]). Error bars represent ± the SD.

**FIG 2:**
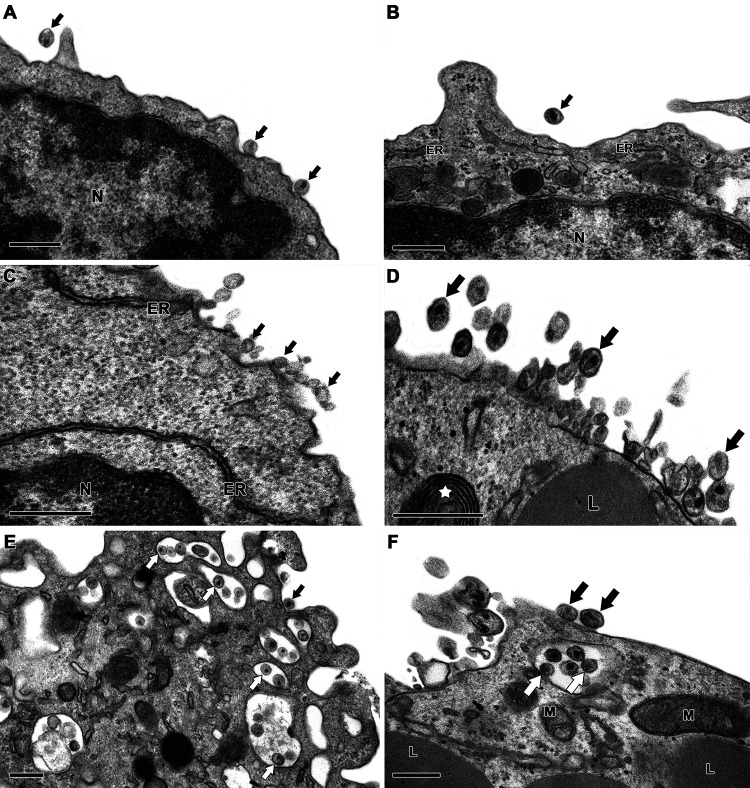
Ultrastructural evaluation of the effect of IL-27 on HIV-1-infected cells. (A and B) HIV-1-infected PBMCs show virus particles at the cell surface (black arrows). (C to F) Addition of IL-27 to HIV-1-infected PBMCs lead to a remarkable accumulation of viral particles adjacent to the infected PBMCs and viral distribution at the cell surface (black arrows) and inside cytosolic vesicles (white arrows). The nucleus (N), endoplasmic reticulum (ER), mitochondria (M), lipid droplets (L), and cytosolic concentric membrane structure (white star) are indicated. Cells were prepared for TEM 48 h after infection, and panels show representative images from one donor out of three. Bars, 0.25 μm (*n* = 3).

Considering that IL-27 has been considered a potential therapeutic agent for HIV-1 infection (26), we aimed to provide additional findings on the ability of IL-27 to regulate HIV-1 infection in PBMCs. Like other authors (16, 20), we initially found that IL-27 reduced HIV-1 replication by 60 to 70% when added to infected PBMCs immediately after infection (see Fig. S1 in the supplemental material). Next, based on reports showing that IL-27 activates several ISGs in uninfected cells (20, 21), we assessed whether this cytokine regulates the expression of the BST-2/Tetherin gene and the activity of the corresponding protein in HIV-1-infected PBMCs. It is well known that BST-2/Tetherin prevents the release of budding HIV-1 virions, thus inducing viral particle accumulation at the infected cell membrane and virion internalization, which eventually inhibits HIV-1 production (24). We detected that BST-2/Tetherin gene expression was significantly upregulated in the infected PBMCs treated with IL-27 (Fig. 1A), and this upregulation was accompanied by an augmentation in the number of CD3^+^ CD4^+^ BST-2/Tetherin^+^ cells in culture (Fig. 1B to E). Of note, IL-27 did not change the expression of the ISGs APOBEC3F and APOBEC3G in the PBMCs (see Fig. S2). We also observed, using transmission electron microscopy (TEM), that few virions were present near the infected cells maintained only with culture medium (Fig. 2A and B), whereas IL-27 promoted accumulation of HIV-1 particles adjacent to the infected PBMCs (Fig. 2C and D). The micrographs show the typical formations of virions attached to each other by BST-2/Tetherin molecules (Fig. 2C and D), according to the model previously proposed by other authors (23, 27). The accumulated virions were reinternalized by infected cells treated with IL-27 (Fig. 2E and F), a consequence of the inability of the virus to completely bud from the infected cell. Our results suggest that BST-2/Tetherin is an essential component of the ability of IL-27 to reduce HIV-1 production in PBMCs.

### BST-2/Tetherin contributes to the IL-27-induced suppression of HIV-1 production when this cytokine is added to infected cells 2 h after infection.

**FIG 3:**
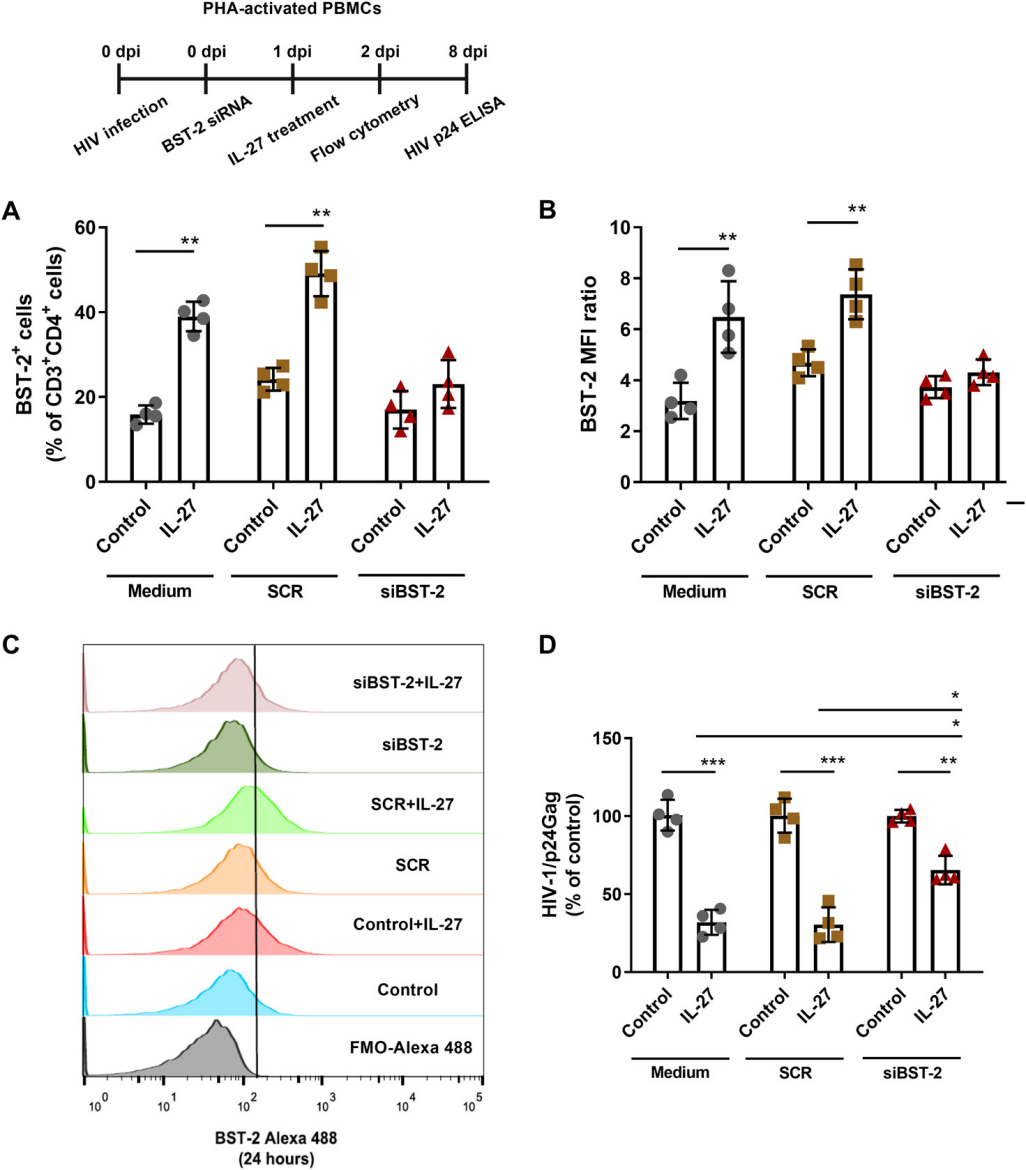
BST-2/Tetherin contributes to the IL-27-induced suppression of HIV-1 production when the cytokine is added to infected cells just after infection. (A to D) HIV-1-infected PBMCs underwent electroporation with a BST-2/Tetherin siRNA (siBST-2) or a scrambled siRNA (SCR) 2 h after infection and, after 24 h, were treated with IL-27 (100 ng/mL). After 24 h of treatment (A and B), BST-2/Tetherin expression was analyzed by flow cytometry within the CD3^+^CD4^+^ gate. (C) Representative histogram of the BST-2/Tetherin expression in cells exposed to IL-27 for 24 h. (D) After 8 days of infection, cell supernatants were collected, and HIV-1 replication was evaluated by measuring the concentration of HIV-1 p24 antigen by enzyme-linked immunosorbent assay (ELISA). The median fluorescence intensity (MFI) ratio was calculated as follows: MFI ratio = specific MFI/FMO MFI. *, *P* < 0.05; **, *P* < 0.01; ***, *P* < 0.001 (two-way ANOVA with Tukey’s multiple-comparison test; *n* = 4; error bars represent ± the SD).

To further support our findings that BST-2/Tetherin participates in this IL-27-mediated anti-HIV-1 effect, we silenced the expression of the *BST2* gene in HIV-1-infected PBMCs. We found that *BST2* silencing 2 h after infection abolished the IL-27-mediated increase in the expression of BST-2/Tetherin (Fig. 3A to C) and, in addition, weakened the anti-HIV-1 effect of IL-27 (Fig. 3D). Altogether, our results show that IL-27 is able to inhibit HIV-1 production when added to PBMCs just after infection and that the innate restriction factor BST-2/Tetherin is a critical mediator of this anti-HIV-1 effect.

### IL-27 favors HIV-1 infection when added 4 days later to infected cells and this effect involves BST-2/Tetherin.

**FIG 4:**
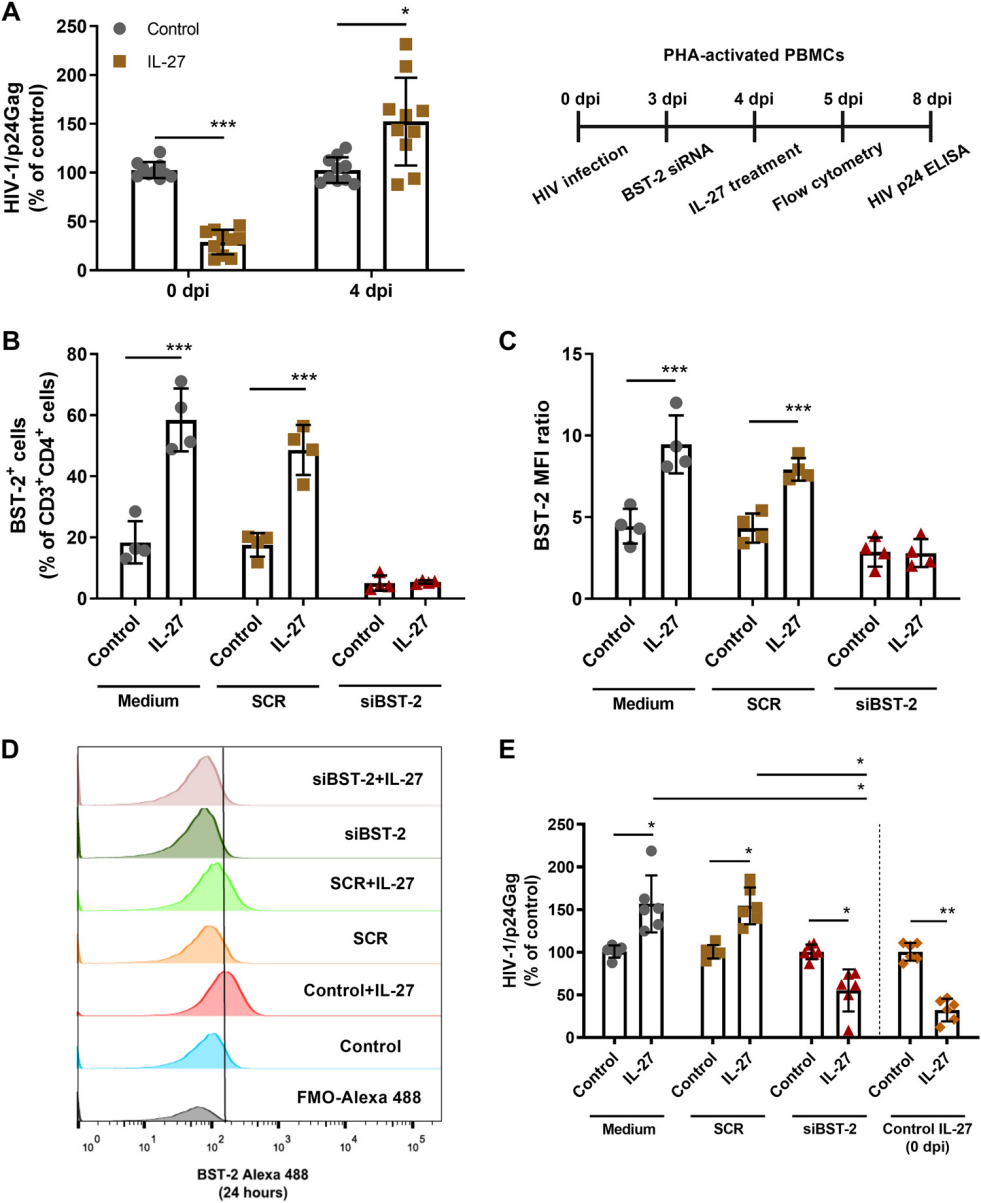
IL-27 favors HIV-1 infection when added later to infected cells, and this effect involves BST-2/Tetherin. (A) HIV-1-infected PBMCs were treated with IL-27 (100 ng/mL) immediately after infection (0 dpi) or 4 days after infection (4 dpi). After 8 days of infection, the cell supernatants were collected, and viral replication was measured by ELISA. (B to E) HIV-1-infected PBMCs underwent electroporation with a BST-2/Tetherin siRNA (siBST-2) or a scrambled siRNA (SCR) 3 days after infection and, after 24 h, were treated with IL-27 (100 ng/mL). After 24 h of treatment (B and C), BST-2/Tetherin expression was analyzed by flow cytometry within the CD3^+^CD4^+^ gate. (D) Representative histogram of BST-2/Tetherin expression in cells exposed to IL-27 for 24 h. (E) After 8 days of infection, cell supernatants were collected, and HIV-1 replication was evaluated by measuring the concentration of HIV-1 p24 antigen by ELISA. The MFI ratio was calculated as follows: MFI ratio = specific MFI/FMO MFI. *, *P* < 0.05; **, *P* < 0.01; ***, *P* < 0.001 (two-way ANOVA with Tukey’s multiple-comparison test; *n* = 10 [A], *n* = 4 [B to G], and *n* = 6 [H]; error bars represent ± the SD).

Because HIV-1-infected cells are continuously exposed to a number of inflammatory mediators in the tissue microenvironment, regardless of how long they have been infected, we investigated whether IL-27 would keep its anti-HIV-1 effect if added later during the course of infection. We found that IL-27 enhanced HIV-1 growth when added on the fourth day after infection (Fig. 4A) as observed from a time course assay (see Fig. S3), contrasting with the HIV-1 inhibition when added to infected PBMCs 2 h after infection. We evaluated the role of BST-2/Tetherin in this enhancing effect by silencing the *BST2* gene in PBMCs infected with HIV-1 for 3 days that were then exposed to IL-27 the next day. As before, *BST2* silencing prevented the IL-27-mediated increase of BST-2/Tetherin expression (Fig. 4B to D) and, notably, abolished the positive effect of IL-27 on HIV-1 growth (Fig. 4E). In fact, silencing the BST-2/Tetherin gene permitted IL-27 to function again as a potent HIV-1 inhibitory factor, similar to when *BST2*-sufficient infected PBMCs were exposed to this cytokine shortly after infection. In conjunction, our results show that IL-27 can promote opposing effects on HIV-1 production in PBMCs and that the molecule BST-2/Tetherin is implicated in these contrasting actions.

### The divergent effects of IL-27 on HIV-1 infection are associated with the dynamic of viral production by infected cells and dependent on cell-to-cell contact.

Taking into account the distinct effects of IL-27 on HIV-1 replication when added immediately after infection (day 0) or 4 days postinfection (dpi) and that up to 60% of the HIV-1 infection in culture is derived from cell-to-cell transmission (28), we pondered wether such divergence could be due to the fact that, when added at 4 dpi, IL-27 would act in a culture containing a larger number of infected cells and, consequently, in an enlarged area of contact between infected and uninfected cells. Moreover, the induction of BST-2/Tetherin expression in this context would result in increased HIV-1 spreading and production, since the inhibition of cell-free virus transmission would be overcome by an enhanced cell-to-cell transmission.

**FIG 5:**
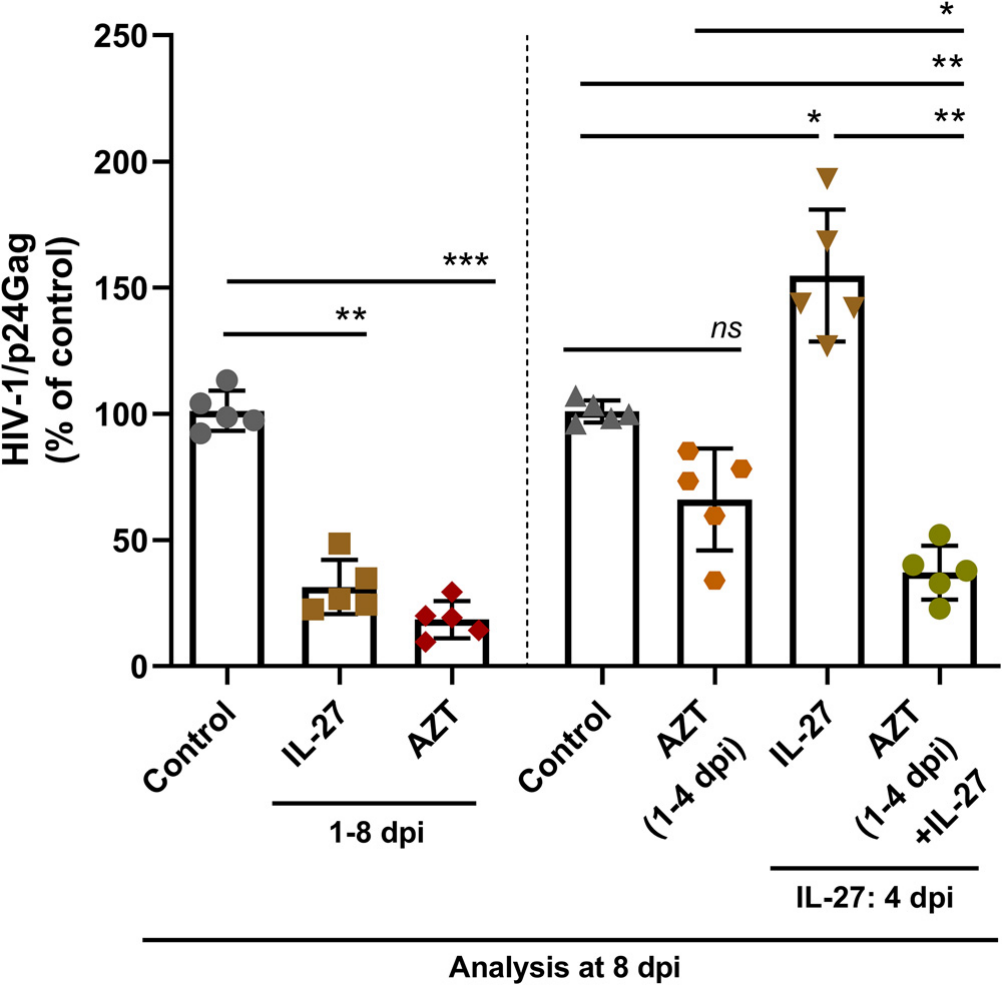
BST-2/Tetherin-mediated contrasting effects of IL-27 on HIV-1 infection are associated with the dynamic of viral production by target cells. PBMCs were infected, treated with IL-27 (100 ng/mL) immediately after infection or at 4 dpi, and maintained in culture for 8 days. HIV-infected PBMCs were maintained in culture the absence (Control) or presence of the reverse transcriptase inhibitor zidovudine (AZT; 1 μM) as indicated, and IL-27 was then added. Infected cells were cultured for more 4 days in the absence of AZT. At the end of the culture period, supernatants were collected, and viral replication was evaluated by measuring the concentration of HIV-1 p24 antigen by ELISA. *, *P* < 0.05; **, *P* < 0.01; ***, *P* < 0.001 (two-way ANOVA with Sidak’s multiple-comparison test; *n* = 5; error bars represent ± the SD).

To evaluate the impact of the infection levels on the divergent effects of IL-27 on HIV-1 production, infected PBMCs were maintained in culture for 4 days in the presence of the reverse transcriptase inhibitor zidovudine (AZT) to reduce HIV-1 replication and viral transmission between cells (29–31). IL-27 was then added, and virus production was measured at 8 dpi. We found that IL-27 did not favor HIV-1 production by the cells maintained with AZT during the first 4 days of culture, whereas addition of IL-27 enhanced HIV-1 production by the cells maintained in culture medium only, as seen before (Fig. 5, two bars on the right). This result strengthened the suggestion that the contrasting action of IL-27 on HIV-1 replication could be dependent on the level of infection dissemination in the culture, which is consequent to cell-free and cell-to-cell viral transmission events.

**FIG 6:**
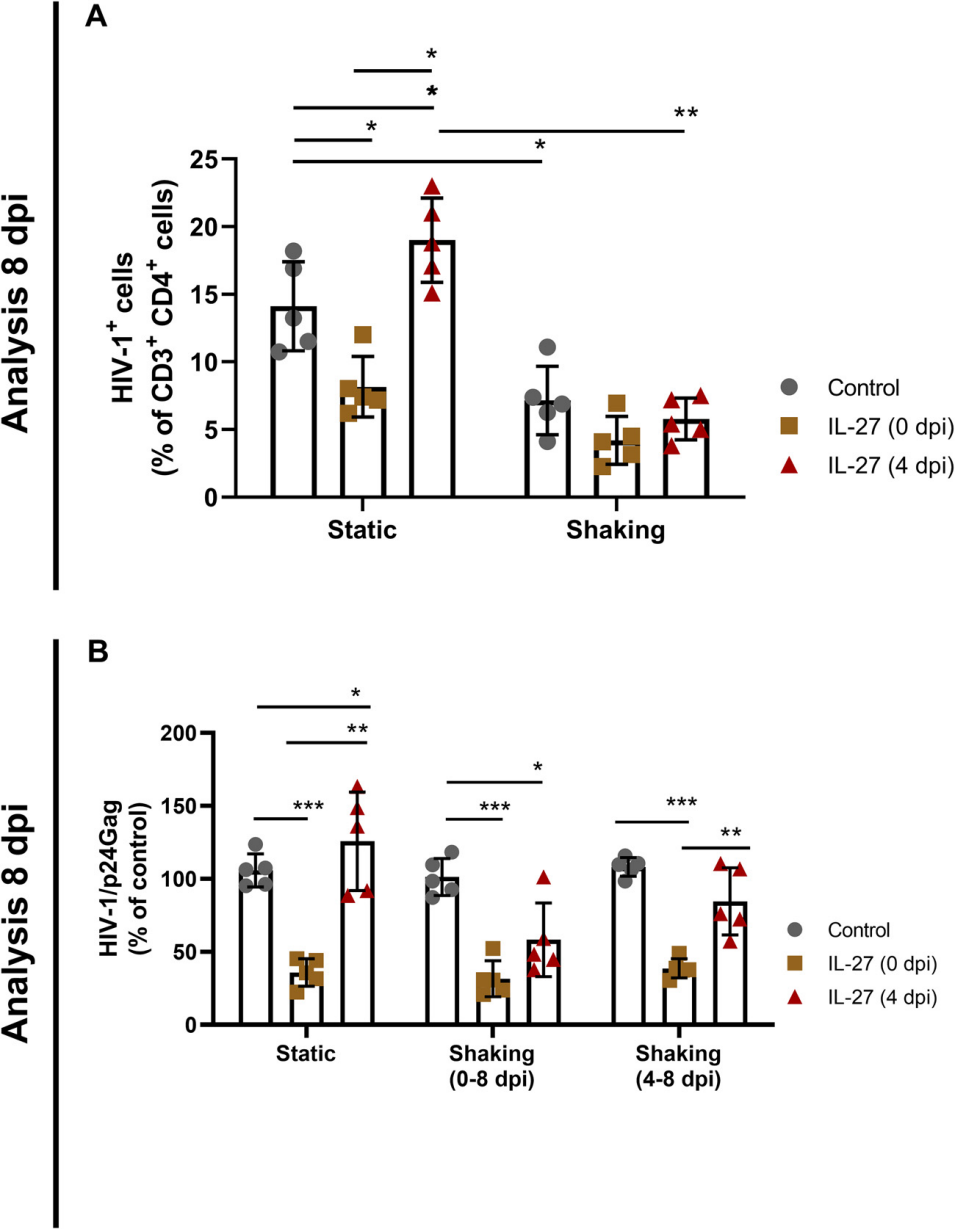
BST-2/Tetherin-mediated contrasting effects of IL-27 on HIV-1 infection are dependent on cell-to-cell contact. PBMCs were infected with HIV-1 and treated with IL-27 (100 ng/mL) immediately after infection (0 dpi) or 4 days later (4 dpi) and maintained under static or shaking culture conditions 8 days (A and B); also, in panel B, cells were maintained under static culture conditions for the first 4 days, followed by shaking for the next 4 days (right group of bars). HIV-1 infection was analyzed by flow cytometry and by ELISA at 8 days postinfection (8 dpi [A and B]). Data show the positive cell population for HIV-1 (A). Viral replication was evaluated by measuring the concentration of HIV-1 p24 antigen by ELISA (B). *, *P* < 0.05; **, *P* < 0.01; ***, *P* < 0.001 (two-way ANOVA with Tukey’s multiple-comparison test; *n* = 5; error bars represent ± the SD).

Therefore, to define the influence of these events of transmission on the outcome of IL-27 treatment, we analyzed whether maintaining the culture under agitation, a condition that inhibits cell-to-cell transmission (32), could prevent the enhancement of HIV-1 replication observed when IL-27 is added at day 4 postinfection. In the static condition, IL-27 treatment at 4 dpi increased the number of HIV-1-infected CD4^+^ cells relative to control and IL-27 treatment at 0 dpi (Fig. 6A, static), but this augmentation did not occur when cells were maintained in constant agitation (Fig. 6A, shaking). At 8 dpi, we found that IL-27 lost its ability to favor HIV-1 growth when added later (4 dpi) to infected cells kept in agitation, recovering its inhibitory effect on HIV-1 replication (Fig. 6B, shaking 0 dpi). We also detected that when both agitation and IL-27 treatment were initiated on 4 dpi, IL-27 did not enhance HIV-1 replication, and the recovering of its inhibitory effect was less pronounced compared to shaking at 0 dpi (Fig. 6B, shaking 4 dpi).

**FIG 7:**
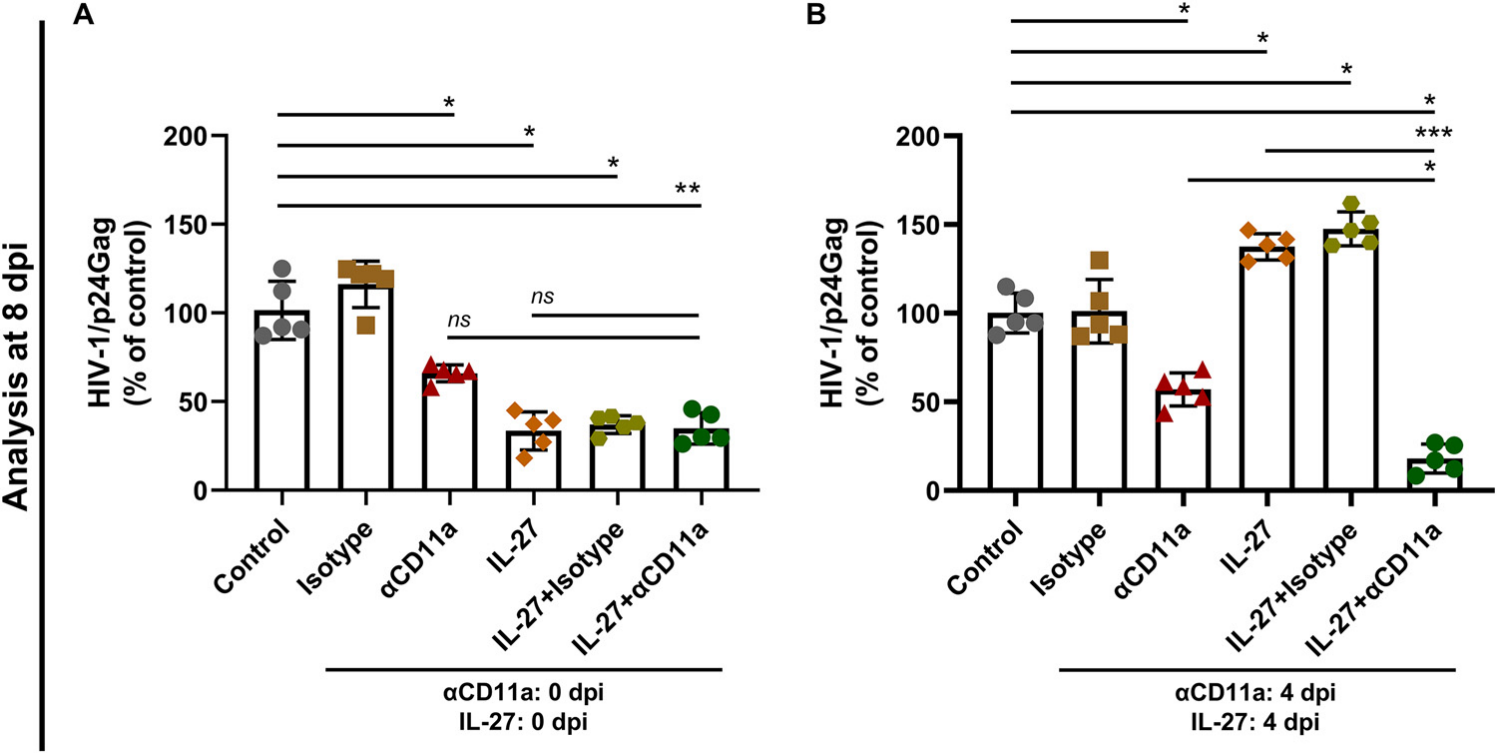
Involvement of CD11a in the effect of IL-27 on HIV-1 infection. (A and B) PBMCs were infected with HIV-1 and treated with IL-27 (100 ng/mL) and a CD11a neutralizing antibody (5 μg/mL) immediately after infection (0 dpi) (A) or 4 days later (4 dpi) (B). Viral replication was quantified 8 days later by measuring the concentration of HIV-1 p24 antigen in the cell culture supernatants by ELISA. *, *P* < 0.05; **, *P* < 0.01; ***, *P* < 0.001 (one-way ANOVA with Tukey’s multiple-comparison test, *n* = 5; error bars represent ± the SD).

Upon identifying that cell-to-cell contact was determinant for the IL-27-induced enhancement of HIV-1 at 4 dpi, we evaluated whether CD11a could be involved in this phenomenon. CD11a is an integrin widely expressed in lymphocytes (33) that contributes to cell-to-cell spread through the formation of virological synapses (34–37). We initially observed that neither HIV-1 infection nor IL-27 exposure modulated the expression of CD11a (see Fig. S5). Then, when cells were maintained in the presence of a CD11a-neutralizing antibody, IL-27 recovered its inhibitory effect when added at 4 dpi, reducing HIV-1 replication to the same level as that found when cells were treated at 0 dpi (Fig. 7). Overall, results from the assays that disturbed the cell-to-cell transmission of HIV-1 show that the dynamic of viral propagation in cultured PBMCs may influence the resultant vector of IL-27 modulation of HIV-1 replication, with participation of BST-2/Tetherin, since either effect, inhibition or increase of HIV-1 replication induced by IL-27, was impaired when this protein was silenced (Fig. 3 and Fig. 4).

## DISCUSSION

**FIG 8:**
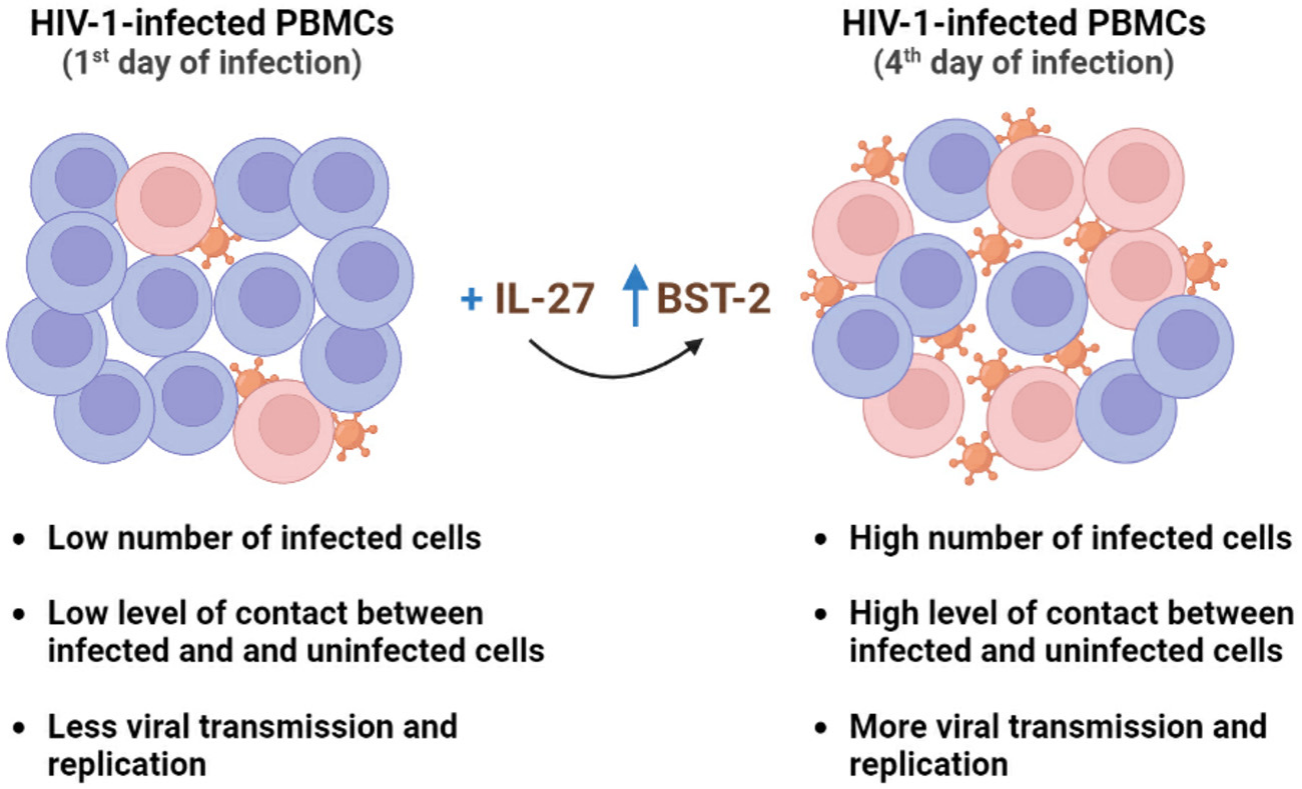
Graphical summary of study data. Induction of BST-2/Tetherin by IL-27 in HIV-1-infected PBMCs leads to divergent outcomes depending on the time of addition of this cytokine. The image was created with BioRender.com.

Our present results add new evidence that IL-27 does not function solely as an HIV-1 restriction factor and, instead, can also act to favor viral propagation, findings that may impact the perspectives of using this cytokine to treat HIV-1 infection. Further studies are needed to elucidate whether IL-27 exhibits similar divergent roles in other HIV-1 target cells, such as macrophages and dendritic cells, and if so, to search for the contribution of BST-2/Tetherin in those infection settings. Remarkably, the contrasting effects of IL-27 on HIV-1 growth in PBMCs are mediated by the same molecule, the IFN-induced protein BST-2/Tetherin. Our findings are summarized in the model presented in Fig. 8.

Initially, BST-2/Tetherin was described as an HIV-1 restriction factor that interferes with the budding and clearance of the virus at the membrane of infected cells (23). However, the consequence of this action is still debated, since several studies have noted differences in the BST-2 viral restraint capacity between HIV-1-infected macrophages, dendritic cells, and lymphocytes (25, 38–41). The most conflicting data show that, in lymphocytes, the “tethering” action of BST-2/Tetherin potentially limits the transmission of cell-free virus in culture but can facilitate cell-to-cell transmission through the accumulation of viral particles at the infected cell membrane, thus leading to the formation of virological synapses (25).

We believe that the recovering of the anti-HIV-1 effect of IL-27 when BST-2/Tetherin is silenced at 4 dpi treatment can be accounted to other HIV-1-inhibitory mechanisms induced by IL-27, which, before surpassed by the pro-HIV-1 action of BST-2/Tetherin, now can act to inhibiting HIV-1 replication. Among them, we quote the cytokine IL-10, which expression is induced by IL-27 in CD4^+^ T lymphocytes (42–45) and can function as an HIV-1 inhibitory molecule, as reported by us and other authors (46–50). In fact, we found that IL-27 upregulated the IL-10 secretion in PBMCs and that neutralization of IL-10 signaling diminished the HIV-1 inhibitory effect of IL-27 (see Fig. S6). The participation of other viral restriction factors, such as MX-1, OAS, and APOBEC3 family members, in IL-27-induced HIV-1 inhibition could be expected; however, their induction by IL-27 seems to be restricted to macrophages, as reported by Imamichi’s group (17, 20), whose findings agree with our results concerning absence of APOBEC3F and APOBEC3G modulation by IL-27 in lymphocytes (see Fig. S2). Interestingly, Wahl et al. (19) showed that HIV-1-infected CD4^+^ T cells can express APOBEC3A upon IL-27 exposure, which has been, until now, the only APOBEC3 family protein known to be modulated in T lymphocytes by IL-27. Finally, macrophages differentiated in the presence of IL-27 present, upon maturation, elevated levels of antiviral microRNAs and reduction of βI-spectrin, a cytoskeletal peptide that functions as an HIV-1 dependency factor (18, 51). Thus, it is up to question if CD4^+^ T cells exposed to or activated in the presence of IL-27 can provide the same results.

The difference in the effects of IL-27 observed between shaking and static conditions can be explained by the reduction of cell-to-cell contact in shaking condition. Thus, we hypothesize that most tethered virions are kept in the infected cells, and eventually recaptured (23), as opposed to a possible viral transference to new target cells when culture is maintained in static condition. Therefore, we propose that to function as an HIV-1 inhibitory factor in PBMCs, IL-27 requires that the number of infected cells should be small at the moment of its addition to infected cells, thus permitting BST-2/Tetherin to act as a restriction factor during the very first rounds of virus production. Also, we verified that the neutralization of CD11a, a key component of HIV-1 virological synapse (34–37), promoted the rescue of the HIV-1 inhibitory potential of IL-27, strengthening cell-to-cell transmission as the main mechanism involved in the HIV-1 propagation in cell culture. More studies are required to explore the possible involvement of IL-27 in HIV-1 transmission through virological synapses since, in the lymphoid tissue, HIV-1 propagation through cell-to-cell contact is expected to be predominant (52, 53).

Our findings that BST-2/Tetherin mediates the opposing effects of IL-27 on HIV-1 production by PBMCs are in agreement with those described by Jolly et al. (25), who found that the cell-to-cell spread of HIV-1 between primary CD4^+^ T cells is more efficient after type 1 IFN treatment, and that this type of HIV-1 dissemination is reduced following the knockdown of BST-2/Tetherin gene expression. In addition, our results are somewhat similar to those reported by Vendrame et al. (54), who observed that IFN-α is less efficient in countering HIV-1 cell-to-cell transmission when added at later times postinfection, or when a higher number of infected cells are present in the culture. IL-27 and IFN-α are intimately connected, as reported by Wahl et al. (19) and, together with BST-2/Tetherin and several other restriction factors, are the core of the innate immune response to HIV-1 (55). However, in more recent years, the canon regarding the beneficial effects of type 1 IFNs during HIV-1 infection has been questioned (56–60). The same scenario could be possible for IL-27, since Ruiz-Riol et al. (61) reported that elevated plasma levels of IL-27 and high expression of the subunit alpha of its receptor in PBMCs are correlated with increased viremia, high loads of HIV-1 provirus and lower HIV-1-specific T-cell response in patients. Because it is not clear whether HIV-1 infection impacts the plasma levels of IL-27 (14, 15), we may ponder that the potential beneficial effects of IL-27 on controlling viral loads in the initial course of an established infection could be jeopardized during the chronic phase, when an elevated number of infected cells are exposed to IL-27 and thus producing more viruses.

Our present work, together with previous data, shed light on the complexity of the HIV-1–host interactions, since the same factors that promote the innate control of HIV-1 could, in the course of infection, become detrimental to the host, through hindering the immune response and favoring viral dissemination. The identification of new interactions between components of the innate immunity, including the type 1 IFN response, could help to elucidate the dynamics of HIV-1 infection, thus contributing to improve the strategies to control viral propagation and disease progression.

## MATERIALS AND METHODS

### Cells, HIV-1 isolates, reagents, and ELISA kits.

PBMCs from healthy donors were obtained by density gradient centrifugation (Ficoll-Paque Premium; GE Healthcare Life Sciences) from buffy coat preparations. These cells were resuspended in RPMI 1640 medium (LGC Bio, São Paulo, Brazil) supplemented with 10% heat-inactivated fetal bovine serum (Cultilab, Brazil), 100 U/mL penicillin, 100 mg/mL streptomycin, 2 mM glutamine, and 10 mM HEPES, stimulated with 5 μg/mL phytohemagglutinin (PHA; Sigma-Aldrich) for 2 to 3 days, and further maintained in culture medium containing 5 U/mL recombinant human Interleukin-2 (Sigma-Aldrich). PBMC infection assays were performed with the HIV-1 R5-isolate Ba-L (donated by the NIH AIDS Research and Reference Reagent Program, Division of AIDS, NIAID, NIH, Bethesda, MD). The virus isolates were expanded in phytohemagglutinin-activated PBMCs from normal donors, as described elsewhere (62). Recombinant human IL-27 and human IL-10 ELISA were purchased from R&D Systems, the antiretroviral drug zidovudine (AZT) was purchased from Sigma-Aldrich, HIV-1 p24 ELISA kits were acquired from Sino Biological, Human IL-10 ELISA was acquired from R&D, and neutralizing antibodies for CD11a and IL-10R, as well their respective isotypes, were purchased from Thermo Fisher and Abcam, respectively. Of note, cellular viability was evaluated throughout the study by trypan blue exclusion assay and by the XTT method, as described previously (50; data not shown).

### HIV-1 infection and the effects of IL-27 on HIV-1 replication.

PBMCs were infected by exposing them to cell-free virus suspensions containing 5 to 10 ng/mL p24 antigen as we described previously (63). After 2 h, the cells were washed to remove excess virus, and culture medium was added to the infected PBMCs. HIV-1 replication was evaluated by measuring the concentration of HIV-1 p24 antigen in the cell culture supernatants by a commercial ELISA kit (Sino Biological), according to the manufacturer’s instructions. To address the role of IL-27 in HIV-1 replication, the HIV-1-infected PBMCs were distributed in 96-well culture plates (2 × 10^5^/well/200 μL) and treated with recombinant human IL-27 at 0 or 4 dpi, without subsequent additions. In some experiments, IL-27 was added to PBMCs over 24 h, and the cells were washed and then infected with HIV-1 as described above. The cells were maintained in culture for different lengths of time, and HIV-1 replication was measured as described above. In specific experiments, the cells were distributed in 6-well culture plates (2 × 10^6^/well/2 mL), placed on an agitator (Bonther, Brazil) in a 37°C/5% CO_2_ incubator, and gently shaken at 40 movements per min for different lengths of time.

### Evaluation of APOBEC and BST-2/Tetherin gene expression by qPCR.

PBMCs were left untreated or treated with IL-27 (100 ng/mL) or IFN-α (10 ng/mL) and, after 18 h, the cells underwent RNA extraction using the RNeasy minikit (Qiagen) according to the manufacturer’s instructions. Total RNA was reverse transcribed with Superscript II according to the manufacturer’s instructions (Invitrogen) in a 25-μL reaction volume, and the resulting cDNA was amplified using SYBR green Master Mix with the following primers: BST-2/Tetherin, F-5′-CTGCAACCACACTGTGAT-3′ and R-5′-ACGCGTCCTGAAGCTTAT-3′; APOBEC-3G, F-5′-GGCTCCACATAAACACGGTTT-3′ and R-5′-AAGGGAATCACGTCCAGGAA-3′; APOBEC-3F, F-5′-TGGAAGTTGTAAAGCACCACTCA-3′ and R-5′-AGCACCTTTCTGCATGACAATG-3′; and HPRT-1, F-5′-CCTGGCGTCGTGATTAGTG3′ and R-5′-TCGAGCAAGACGTTCAGTCC-3′’. The HPRT gene was used as housekeeping control. Reactions were run in triplicate using a StepOne Plus instrument (Applied Biosystems) for all independent experiments.

### Analysis of cell markers by flow cytometry.

At the indicated times, cells were removed from culture flasks, incubated with AB+ human serum and mouse serum for 15 min (blocking solution: phosphate-buffered saline plus 2 mM EDTA, 1% bovine serum albumin, 25% human serum, and 25% mouse serum), labeled with specific antibodies for 40 min, and permeabilized for intracellular staining (when using an anti-HIV-1 antibody). All staining incubations were performed using the blocking solution as the diluent. The following antibodies, as well as the corresponding isotype controls, were used: mouse anti-human CD3-APC (BD Biosciences), mouse anti-human CD4-APC-Cy7 (BD Biosciences), mouse anti-human CD317 (BST2/PDCA-1)-Alexa Fluor 488 (eBioscience), anti-HIV-1 gag (RD1, KC57 clone; Beckman Coulter), and mouse anti-human CD11a-Pe-Cy5 (BD Biosciences). After a washing step, the cells were fixed, and the staining was evaluated with a FACSCelesta flow cytometer (BD Biosciences) equipped with FACSDiva software (BD Biosciences). All analyses were performed after doublet and debris exclusion using forward versus side scatter parameters, and live/dead exclusion was performed (Live/Dead Fixable Scarlet 723 stain kit; Thermo Fisher, USA). HIV-1, BST-2/Tetherin, and CD11a expression were analyzed based on FMO (fluorescence minus one) controls. The FMO controls and the HIV-1 negative control are depicted in Fig. S7. Figures S8 and S9 in the supplemental material show the representative histograms of experiments. The median fluorescence intensity (MFI) ratio was calculated as follows: MFI ratio = specific MFI/FMO MFI. The data were analyzed by using FlowJo software (TreeStar).

### Ultrastructural analysis of the effects of IL-27 on virus release from infected cells.

For TEM analysis, HIV-1-infected PBMCs treated or not with 100 ng/mL of IL-27 during 48 h were fixed in 2.5% glutaraldehyde in 0.1 M cacodylate buffer (pH 7.2) for 1 h at 25°C and postfixed with a solution of 1% OsO_4_ containing 0.8% potassium ferricyanide and 2.5 mM CaCl_2_ in the same buffer for 20 min at 25°C. Then, the samples were dehydrated in an ascending acetone series and embedded in Polybed 812 resin (Polysciences, USA). Ultrathin sections were obtained, stained with uranyl acetate and lead citrate, and examined with the transmission electron microscope JEM 1011 (Jeol, Tokyo, Japan) at the Fiocruz Electronic Microscopy Platform.

### BST-2/Tetherin gene silencing.

HIV-1-infected PBMCs underwent BST-2/Tetherin siRNA electroporation 2 h (protocol 1) or 3 days (protocol 2) after infection. A total of 10^7^ PBMCs were pelleted and resuspended in 100 μL of prewarmed electroporation buffer [in-house prepared buffer solution 3P containing 5 mM KCl, 15 mM MgCl_2_, 90 mM NaCl, 10 mM glucose, 0.4 mM Ca(NO_3_)_2_, and 40 mM Na_2_HPO_4_/NaH_2_PO_4_ (pH 7.2) (64)] premixed with BST-2/siRNA (5′-AAGCGTGAGAATCGCGGACAA; NM_004335; Hs_BST2_5 FlexiTube siRNA; Qiagen, catalog number SI02777054) or scramble/siRNA (Ctrl_AllStrar_1; Qiagen, catalog number SI03650318) to a final concentration of 200 nM. Then, the cells were transferred to sterile 0.2-cm generic cuvettes (Mirus Biotech, catalog number MIR 50121) and electroporated with a Nucleofector 2b device (T-020 program; Lonza). After electroporation, the cells were immediately and gently resuspended in 1 mL of prewarmed RPMI medium supplemented with 5 U/mL rhIL-2 (Sigma-Aldrich) and 20% fetal bovine serum, transferred to cell culture flasks, and incubated at 37°C in 5% CO_2_ for 24 h. The day after electroporation, the cells were centrifuged and divided into two groups, one with and one without rhIL-27. The cells were maintained in culture for an additional 24 or 48 h for flow cytometry analysis or for an additional 5 days (protocol 1) or 8 days (protocol 2) to measure HIV-1 replication.

### Statistical analysis.

All results presented in this study were prepared using Prism 9.0 software (GraphPad, San Diego, CA). Statistical analysis was performed using a Student *t* test or one-way analysis of variance (ANOVA) or two-way ANOVA, followed by a Sidak’s or Tukey multiple-comparison test when appropriated. The results are shown as means ± the standard deviations (SD), and comparisons between values were considered significantly different when the *P* value was <0.05 (*, *P ≤ *0.05; **, *P ≤ *0.01; ***, *P ≤ *0.001).

### Statement of ethics.

Experimental procedures involving human cells from healthy donors were performed with samples obtained after we obtained written informed consent and were approved by the Institutional Review Board of the Oswaldo Cruz Institute/Fiocruz (Rio de Janeiro, Brazil) under number 397-07.

## References

[1] Pflanz S, Timans JC, Cheung J, Rosales R, Kanzler H, Gilbert J, Hibbert L, Churakova T, Travis M, Vaisberg E, Blumenschein WM, Mattson JD, Wagner JL, To W, Zurawski S, McClanahan TK, Gorman DM, Bazan JF, de Waal Malefyt R, Rennick D, Kastelein RA. 2002. IL-27, a heterodimeric cytokine composed of EBI3 and p28 protein, induces proliferation of naive CD4^+^ T cells. Immunity 16:779–790. 10.1016/s1074-7613(02)00324-2. 12121660

[2] Pflanz S, Hibbert L, Mattson J, Rosales R, Vaisberg E, Bazan JF, Phillips JH, McClanahan TK, de Waal Malefyt R, Kastelein RA. 2004. WSX-1 and glycoprotein 130 constitute a signal-transducing receptor for IL-27. J Immunol 172:2225–2231. 10.4049/jimmunol.172.4.2225. 14764690

[3] Villarino A, Huang E, Hunter CA. 2004. Understanding the pro- and anti-inflammatory properties of IL-27. J Immunol 173:715–720. 10.4049/jimmunol.173.2.715. 15240655

[4] Hunter CA. 2005. New IL-12-family members: IL-23 and IL-27, cytokines with divergent functions. Nat Rev Immunol 5:521–531. 10.1038/nri1648. 15999093

[5] Stumhofer JS, Silver JS, Laurence A, Porrett PM, Harris TH, Turka LA, Ernst M, Saris CJM, O’Shea JJ, Hunter CA. 2007. Interleukins 27 and 6 induce STAT3-mediated T cell production of interleukin 10. Nat Immunol 8:1363–1371. 10.1038/ni1537. 17994025

[6] Yoshida H, Miyazaki Y. 2008. Regulation of immune responses by interleukin-27. Immunol Rev 226:234–247. 10.1111/j.1600-065X.2008.00710.x. 19161428

[7] Guzzo C, Che Mat NF, Gee K. 2010. Interleukin-27 induces a STAT1/3- and NF-κB-dependent proinflammatory cytokine profile in human monocytes. J Biol Chem 285:24404–24411. 10.1074/jbc.M110.112599. 20519510 PMC2915676

[8] Brenchley JM, Douek DC. 2008. HIV infection and the gastrointestinal immune system. Mucosal Immunol 1:23–30. 10.1038/mi.2007.1. 19079157 PMC2777614

[9] Brenchley JM, Douek DC. 2008. The mucosal barrier and immune activation in HIV pathogenesis. Curr Opin HIV AIDS 3:356–361. 10.1097/COH.0b013e3282f9ae9c. 19372990 PMC2789390

[10] van Grevenynghe J, Halwani R, Chomont N, Ancuta P, Peretz Y, Tanel A, Procopio FA, Shi Y, Said EA, Haddad EK, Sekaly RP. 2008. Lymph node architecture collapse and consequent modulation of FOXO3a pathway on memory T- and B-cells during HIV infection. Semin Immunol 20:196–203. 10.1016/j.smim.2008.07.008. 18757210

[11] Biancotto A, Grivel JC, Iglehart SJ, Vanpouille C, Lisco A, Sieg SF, Debernardo R, Garate K, Rodriguez B, Margolis LB, Lederman MM. 2007. Abnormal activation and cytokine spectra in lymph nodes of people chronically infected with HIV-1. Blood 109:4272–4279. 10.1182/blood-2006-11-055764. 17289812 PMC1885500

[12] Waki K, Freed EO. 2010. Macrophages and cell-cell spread of HIV-1. *In* Viruses 2:1603–1620. 10.3390/v2081603. 21552427 PMC3088113

[13] Felts RL, Narayan K, Estes JD, Shi D, Trubey CM, Fu J, Hartnell LM, Ruthel GT, Schneider DK, Nagashima K, Bess JW, Jr, Bavari S, Lowekamp BC, Bliss D, Lifson JD, Subramaniam S. 2010. 3D visualization of HIV transfer at the virological synapse between dendritic cells and T cells. Proc Natl Acad Sci USA 107:13336–13341. 10.1073/pnas.1003040107. 20624966 PMC2922156

[14] He L, Zhao J, Wang MH, Siu KKY, Gan Y-X, Chen L, Zee BCY, Yang L, Kung H-F, Yang Z-R, He M-L. 2014. Interleukin-27 is differentially associated with HIV viral load and CD4^+^ T cell counts in therapy-naive HIV-mono-infected and HIV/HCV-coinfected Chinese. PLoS One 9:e96792. 10.1371/journal.pone.0096792. 24816922 PMC4016030

[15] Swaminathan S, Hu Z, Rupert AW, Higgins JM, Dewar RL, Stevens R, Chen Q, Rehm CA, Metcalf JA, Baseler MW, Lane HC, Imamichi T. 2014. Plasma interleukin-27 (IL-27) levels are not modulated in patients with chronic HIV-1 infection. PLoS One 9:e98989. 10.1371/journal.pone.0098989. 24896094 PMC4045808

[16] Fakruddin JM, Lempicki RA, Gorelick RJ, Yang J, Adelsberger JW, Garcia-Pineres AJ, Pinto LA, Lane HC, Imamichi T. 2007. Noninfectious papilloma virus-like particles inhibit HIV-1 replication: implications for immune control of HIV-1 infection by IL-27. Blood 109:1841–1849. 10.1182/blood-2006-02-001578. 17068156 PMC1801045

[17] Chen Q, Swaminathan S, Yang D, Dai L, Sui H, Yang J, Hornung RL, Wang Y, Huang da W, Hu X, Lempicki RA, Imamichi T. 2013. Interleukin-27 is a potent inhibitor of cis HIV-1 replication in monocyte-derived dendritic cells via a type I interferon-independent pathway. PLoS One 8:e59194. 10.1371/journal.pone.0059194. 23527130 PMC3604098

[18] Dai L, Lidie KB, Chen Q, Adelsberger JW, Zheng X, Huang D, Yang J, Lempicki RA, Rehman T, Dewar RL, Wang Y, Hornung RL, Canizales KA, Lockett SJ, Lane HC, Imamichi T. 2013. IL-27 inhibits HIV-1 infection in human macrophages by down-regulating host factor SPTBN1 during monocyte to macrophage differentiation. J Exp Med 210:517–534. 10.1084/jem.20120572. 23460728 PMC3600911

[19] Greenwell-Wild T, Vazquez N, Jin W, Rangel Z, Munson PJ, Wahl SM. 2009. Interleukin-27 inhibition of HIV-1 involves an intermediate induction of type I interferon. Blood 114:1864–1874. 10.1182/blood-2009-03-211540. 19556424 PMC2738572

[20] Imamichi T, Yang J, Huang DW, Brann TW, Fullmer BA, Adelsberger JW, Lempicki RA, Baseler MW, Lane HC. 2008. IL-27, a novel anti-HIV cytokine, activates multiple interferon-inducible genes in macrophages. AIDS 22:39–45. 10.1097/QAD.0b013e3282f3356c. 18090390

[21] Guzzo C, Jung M, Graveline A, Banfield BW, Gee K. 2012. IL-27 increases BST-2 expression in human monocytes and T cells independently of type I IFN. Sci Rep 2:974. 10.1038/srep00974. 23240078 PMC3521153

[22] Neil SJ, Sandrin V, Sundquist WI, Bieniasz PD. 2007. An interferon-alpha-induced tethering mechanism inhibits HIV-1 and Ebola virus particle release but is counteracted by the HIV-1 Vpu protein. Cell Host Microbe 2:193–203. 10.1016/j.chom.2007.08.001. 18005734 PMC3793644

[23] Neil SJ, Zang T, Bieniasz PD. 2008. Tetherin inhibits retrovirus release and is antagonized by HIV-1 Vpu. Nature 451:425–430. 10.1038/nature06553. 18200009

[24] van Damme N, Goff D, Katsura C, Jorgenson RL, Mitchell R, Johnson MC, Stephens EB, Guatelli J. 2008. The interferon-induced protein BST-2 restricts HIV-1 release and is downregulated from the cell surface by the viral Vpu protein. Cell Host Microbe 3:245–252. 10.1016/j.chom.2008.03.001. 18342597 PMC2474773

[25] Jolly C, Booth NJ, Neil SJ. 2010. Cell-cell spread of human immunodeficiency virus type 1 overcomes tetherin/BST-2-mediated restriction in T cells. J Virol 84:12185–12199. 10.1128/JVI.01447-10. 20861257 PMC2976402

[26] Swaminathan S, Dai L, Lane HC, Imamichi T. 2013. Evaluating the potential of IL-27 as a novel therapeutic agent in HIV-1 infection. Cytokine Growth Factor Rev 24:571–577. 10.1016/j.cytogfr.2013.07.001. 23962745 PMC3851681

[27] Gupta RK, Towers GJ. 2009. A tail of Tetherin: how pandemic HIV-1 conquered the world. Cell Host Microbe 6:393–395. 10.1016/j.chom.2009.11.002. 19917491 PMC3556580

[28] Iwami S, Takeuchi JS, Nakaoka S, Mammano F, Clavel F, Inaba H, Kobayashi T, Misawa N, Aihara K, Koyanagi Y, Sato K. 2015. Cell-to-cell infection by HIV contributes over half of virus infection. Elife 4. 10.7554/eLife.08150. PMC4592948 26441404

[29] Gupta P, Balachandran R, Ho M, Enrico A, Rinaldo C. 1989. Cell-to-cell transmission of human immunodeficiency virus type 1 in the presence of azidothymidine and neutralizing antibody. J Virol 63:2361–2365. 10.1128/JVI.63.5.2361-2365.1989. 2704079 PMC250658

[30] Puigdomenech I, Massanella M, Cabrera C, Clotet B, Blanco J. 2009. On the steps of cell-to-cell HIV transmission between CD4 T cells. Retrovirology 6:89. 10.1186/1742-4690-6-89. 19825175 PMC2768678

[31] Sigal A, Kim JT, Balazs AB, Dekel E, Mayo A, Milo R, Baltimore D. 2011. Cell-to-cell spread of HIV permits ongoing replication despite antiretroviral therapy. Nature 477:95–98. 10.1038/nature10347. 21849975

[32] Sourisseau M, Sol-Foulon N, Porrot F, Blanchet F, Schwartz O. 2007. Inefficient human immunodeficiency virus replication in mobile lymphocytes. J Virol 81:1000–1012. 10.1128/JVI.01629-06. 17079292 PMC1797449

[33] Verma NK, Kelleher D. 2017. Not just an adhesion molecule: LFA-1 contact tunes the T lymphocyte program. J Immunol 199:1213–1221. 10.4049/jimmunol.1700495. 28784685

[34] Jolly C, Mitar I, Sattentau QJ. 2007. Adhesion molecule interactions facilitate human immunodeficiency virus type 1-induced virological synapse formation between T cells. J Virol 81:13916–13921. 10.1128/JVI.01585-07. 17913807 PMC2168851

[35] Rudnicka D, Feldmann J, Porrot F, Wietgrefe S, Guadagnini S, Prévost M-C, Estaquier J, Haase AT, Sol-Foulon N, Schwartz O. 2009. Simultaneous cell-to-cell transmission of human immunodeficiency virus to multiple targets through polysynapses. J Virol 83:6234–6246. 10.1128/JVI.00282-09. 19369333 PMC2687379

[36] Cicala C, Arthos J, Fauci AS. 2011. HIV-1 envelope, integrins and coreceptor use in mucosal transmission of HIV. J Transl Med 9:S2. 10.1186/1479-5876-9-S1-S2. 21284901 PMC3105502

[37] Starling S, Jolly C. 2016. LFA-1 engagement triggers T cell polarization at the HIV-1 virological synapse. J Virol 90:9841–9854. 10.1128/JVI.01152-16. 27558417 PMC5068534

[38] Casartelli N, Sourisseau M, Feldmann J, Guivel-Benhassine F, Mallet A, Marcelin AG, Guatelli J, Schwartz O. 2010. Tetherin restricts productive HIV-1 cell-to-cell transmission. PLoS Pathog 6:e1000955. 10.1371/journal.ppat.1000955. 20585562 PMC2887479

[39] Chu H, Wang JJ, Qi M, Yoon JJ, Chen X, Wen X, Hammonds J, Ding L, Spearman P. 2012. Tetherin/BST-2 is essential for the formation of the intracellular virus-containing compartment in HIV-infected macrophages. Cell Host Microbe 12:360–372. 10.1016/j.chom.2012.07.011. 22980332 PMC3444820

[40] Cocka LJ, Bates P. 2012. Identification of alternatively translated Tetherin isoforms with differing antiviral and signaling activities. PLoS Pathog 8:e1002931. 10.1371/journal.ppat.1002931. 23028328 PMC3460627

[41] Giese S, Marsh M. 2014. Tetherin can restrict cell-free and cell-cell transmission of HIV from primary macrophages to T cells. PLoS Pathog 10:e1004189. 10.1371/journal.ppat.1004189. 24991932 PMC4081785

[42] de Aquino MT, Kapil P, Hinton DR, Phares TW, Puntambekar SS, Savarin C, Bergmann CC, Stohlman SA. 2014. IL-27 limits central nervous system viral clearance by promoting IL-10 and enhances demyelination. J Immunol 193:285–294. 10.4049/jimmunol.1400058. 24890725 PMC4067872

[43] Chang KK, Liu LB, Jin LP, Zhang B, Mei J, Li H, Wei CY, Zhou WJ, Zhu XY, Shao J, Li DJ, Li MQ. 2017. IL-27 triggers IL-10 production in Th17 cells via a c-Maf/RORγt/Blimp-1 signal to promote the progression of endometriosis. Cell Death Dis 8:e2666. 10.1038/cddis.2017.95. 28300844 PMC5386585

[44] Clement M, Marsden M, Stacey MA, Abdul-Karim J, Gimeno Brias S, Costa Bento D, Scurr MJ, Ghazal P, Weaver CT, Carlesso G, Clare S, Jones SA, Godkin A, Jones GW, Humphreys IR. 2016. Cytomegalovirus-specific IL-10-producing CD4^+^ T cells are governed by type-I IFN-induced IL-27 and promote virus persistence. PLoS Pathog 12:e1006050. 10.1371/journal.ppat.1006050. 27926930 PMC5142785

[45] Murugaiyan G, Mittal A, Lopez-Diego R, Maier LM, Anderson DE, Weiner HL. 2009. IL-27 is a key regulator of IL-10 and IL-17 production by human CD4^+^ T cells. J Immunol 183:2435–2443. 10.4049/jimmunol.0900568. 19625647 PMC2904948

[46] Andrade RM, Lima PG, Filho RG, Hygino J, Milczanowski SF, Andrade AF, Lauria C, Brindeiro R, Tanuri A, Bento CA. 2007. Interleukin-10-secreting CD4 cells from aged patients with AIDS decrease in-vitro HIV replication and tumour necrosis factor alpha production. AIDS 21:1763–1770. 10.1097/QAD.0b013e3282ca83fa. 17690575

[47] Weissman D, Poli G, Fauci AS. 1994. Interleukin 10 blocks HIV replication in macrophages by inhibiting the autocrine. AIDS Res Hum Retroviruses 10:1199–1206. 10.1089/aid.1994.10.1199. 7848677

[48] Temerozo JR, Joaquim R, Regis EG, Savino W, Bou-Habib DC. 2013. Macrophage resistance to HIV-1 infection is enhanced by the neuropeptides VIP and PACAP. PLoS One 8:e67701. 10.1371/journal.pone.0067701. 23818986 PMC3688615

[49] Temerozo JR, de Azevedo SSD, Insuela DBR, Vieira RC, Ferreira PLC, Carvalho VF, Bello G, Bou-Habib DC. 2018. The neuropeptides vasoactive intestinal peptide and pituitary adenylate cyclase-activating polypeptide control HIV-1 infection in macrophages through activation of protein kinases A and C. Front Immunol 9:1336. 10.3389/fimmu.2018.01336. 29951068 PMC6008521

[50] Victoria S, Temerozo JR, Gobbo L, Pimenta-Inada HK, Bou-Habib DC. 2013. Activation of Toll-like receptor 2 increases macrophage resistance to HIV-1 infection. Immunobiology 218:1529–1536. 10.1016/j.imbio.2013.06.006. 23891328

[51] Swaminathan S, Hu X, Zheng X, Kriga Y, Shetty J, Zhao Y, Stephens R, Tran B, Baseler MW, Yang J, Lempicki RA, Huang D, Lane HC, Imamichi T. 2013. Interleukin-27-treated human macrophages induce the expression of novel microRNAs which may mediate anti-viral properties. Biochem Biophys Res Commun 434:228–234. 10.1016/j.bbrc.2013.03.046. 23535375 PMC3700531

[52] Law KM, Satija N, Esposito AM, Chen BK. 2016. Cell-to-cell spread of HIV and viral pathogenesis. Adv Virus Res 95:43–85. 10.1016/bs.aivir.2016.03.001. 27112280 PMC12989982

[53] Bracq L, Xie M, Benichou S, Bouchet J. 2018. Mechanisms for cell-to-cell transmission of HIV-1. Front Immunol 9:260. 10.3389/fimmu.2018.00260. 29515578 PMC5825902

[54] Vendrame D, Sourisseau M, Perrin V, Schwartz O, Mammano F. 2009. Partial inhibition of human immunodeficiency virus replication by type I interferons: impact of cell-to-cell viral transfer. J Virol 83:10527–10537. 10.1128/JVI.01235-09. 19706714 PMC2753145

[55] Rustagi A, Gale M, Jr. 2014. Innate antiviral immune signaling, viral evasion and modulation by HIV-1. J Mol Biol 426:1161–1177. 10.1016/j.jmb.2013.12.003. 24326250 PMC7425209

[56] Soper A, Kimura I, Nagaoka S, Konno Y, Yamamoto K, Koyanagi Y, Sato K. 2017. Type I interferon responses by HIV-1 infection: association with disease progression and control. Front Immunol 8:1823. 10.3389/fimmu.2017.01823. 29379496 PMC5775519

[57] Lehmann C, Lafferty M, Garzino-Demo A, Jung N, Hartmann P, Fatkenheuer G, Wolf JS, van Lunzen J, Romerio F. 2010. Plasmacytoid dendritic cells accumulate and secrete interferon alpha in lymph nodes of HIV-1 patients. PLoS One 5:e11110. 10.1371/journal.pone.0011110. 20559432 PMC2885422

[58] Zhen A, Rezek V, Youn C, Lam B, Chang N, Rick J, Carrillo M, Martin H, Kasparian S, Syed P, Rice N, Brooks DG, Kitchen SG. 2017. Targeting type I interferon-mediated activation restores immune function in chronic HIV infection. J Clin Invest 127:260–268. 10.1172/JCI89488. 27941243 PMC5199686

[59] Cheng L, Yu H, Li G, Li F, Ma J, Li J, Chi L, Zhang L, Su L. 2017. Type I interferons suppress viral replication but contribute to T cell depletion and dysfunction during chronic HIV-1 infection. JCI Insight 2. 10.1172/jci.insight.94366. PMC5470878 28614789

[60] Cheng L, Ma J, Li J, Li D, Li G, Li F, Zhang Q, Yu H, Yasui F, Ye C, Tsao LC, Hu Z, Su L, Zhang L. 2017. Blocking type I interferon signaling enhances T cell recovery and reduces HIV-1 reservoirs. J Clin Invest 127:269–279. 10.1172/JCI90745. 27941247 PMC5199717

[61] Ruiz-Riol M, Berdnik D, Llano A, Mothe B, Galvez C, Perez-Alvarez S, Oriol-Tordera B, Olvera A, Silva-Arrieta S, Meulbroek M, Pujol F, Coll J, Martinez-Picado J, Ganoza C, Sanchez J, Gomez G, Wyss-Coray T, Brander C. 2017. Identification of interleukin-27 (IL-27)/IL-27 receptor subunit alpha as a critical immune axis for *in vivo* HIV control. J Virol 91:e00441-17. 10.1128/JVI.00441-17. 28592538 PMC5533920

[62] Lima RG, van Weyenbergh J, Saraiva EM, Barral-Netto M, Galvao-Castro B, Bou-Habib DC. 2002. The replication of human immunodeficiency virus type 1 in macrophages is enhanced after phagocytosis of apoptotic cells. J Infect Dis 185:1561–1566. 10.1086/340412. 12023761

[63] Regis EG, Barreto-de-Souza V, Morgado MG, Bozza MT, Leng L, Bucala R, Bou-Habib DC. 2010. Elevated levels of macrophage migration inhibitory factor (MIF) in the plasma of HIV-1-infected patients and in HIV-1-infected cell cultures: a relevant role on viral replication. Virology 399:31–38. 10.1016/j.virol.2009.12.018. 20085845 PMC3140709

[64] Chicaybam L, Sodre AL, Curzio BA, Bonamino MH. 2013. An efficient low cost method for gene transfer to T lymphocytes. PLoS One 8:e60298. 10.1371/journal.pone.0060298. 23555950 PMC3608570

