# Supplementary material for: Interleukin-27 Promotes Divergent Effects on HIV-1 Infection in Peripheral Blood Mononuclear Cells through BST-2/Tetherin
Source: J Virol. 2023 Jan 5;97(1):e01752-22. doi: 10.1128/jvi.01752-22 (PMC9888194; doi:10.1128/jvi.01752-22)
Supplement: Supplemental file 1 — Fig. S1 to S9. Download jvi.01752-22-s0001.pdf, PDF file, 0.8 MB [file jvi.01752-22-s0001.pdf]

Supplementary Information

Interleukin 27 promotes divergent effects on HIV-1 infection in peripheral blood mononuclear cells through BST-2/Tetherin

Jairo R. Temerozo[1,5,a,*], Pedro L. C. Ferreira[1,a], , Leandra Linhares-Lacerda[2,5], Rhaíssa C. Vieira[1], Bruno Cister-Alves[1], Livia Gobbo[1], Marcelo Ribeiro-Alves[4,5], Rubem F. S. Menna-Barreto[3], Dumith Chequer Bou-Habib[1,5,*]

[1]Laboratory on Thymus Research, Oswaldo Cruz Institute/Fiocruz, Rio de Janeiro, Brazil; [2]Laboratory of Immunobiology of Leishmaniasis, Department of Immunology, Paulo de Goes Institute of Microbiology, Federal University of Rio de Janeiro, Rio de Janeiro, Brazil; [3]Laboratory of Cellular Biology, Oswaldo Cruz Institute/Fiocruz, Rio de Janeiro, Brazil, [4]HIV/AIDS Clinical Research Center, Evandro Chagas National Institute of Infectology, Fiocruz, Rio de Janeiro, Brazil; [5]National Institute of Science and Technology on Neuroimmunomodulation, Rio de Janeiro, Brazil.

*JRT and PLCF are co-first authors, as they contributed equally to this work.

Corresponding authors: Dumith Chequer Bou-Habib and Jairo R. Temerozo

This PDF file includes:

Figs. S1 to S9

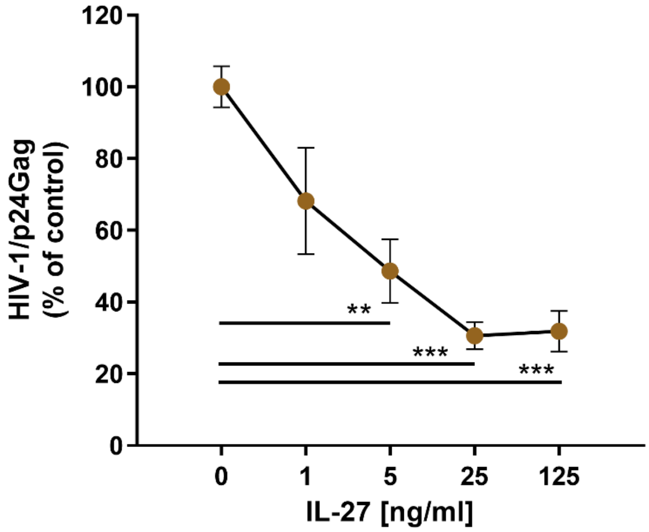

Figure S1. IL-27 inhibits HIV-1 replication in PBMCs. HIV-1-infected PBMCs were treated with different concentrations of IL-27 two hours after infection. Viral replication was quantified 8 days later by measuring the concentration of HIV-1 p24 antigen in the cell culture supernatants by ELISA. **, $p<0.01$; ***, $p<0.001$; One-way ANOVA with Tukey's multiple comparisons test, n=5; error bars represent ± SD (standard deviation).

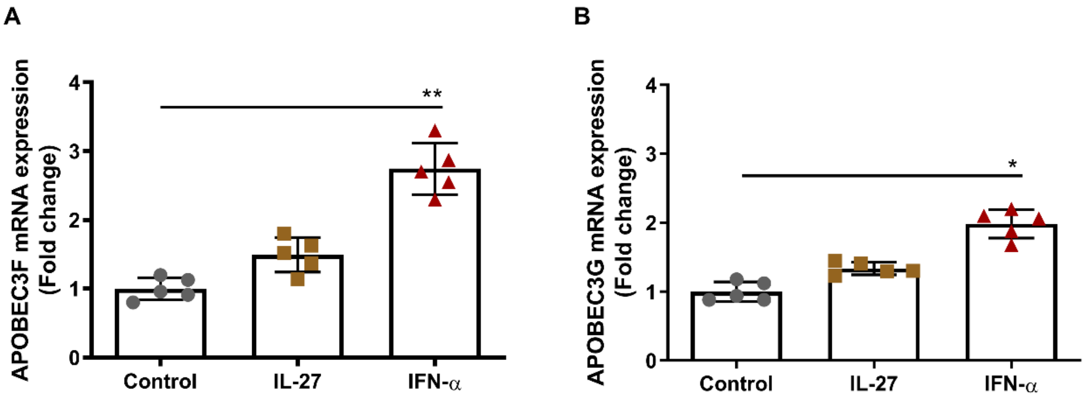

Figure S2. IL-27 does not induce the expression of *APOBEC3F* or *APOBEC3G*. (A, B) PBMCs were exposed to IL-27 (100 ng/ml) or IFN-α (10 ng/ml), and after 18 hours, the expression of *APOBEC3F* (A) and *APOBEC3G* (B) was analyzed by real-time PCR. n=5 (A).

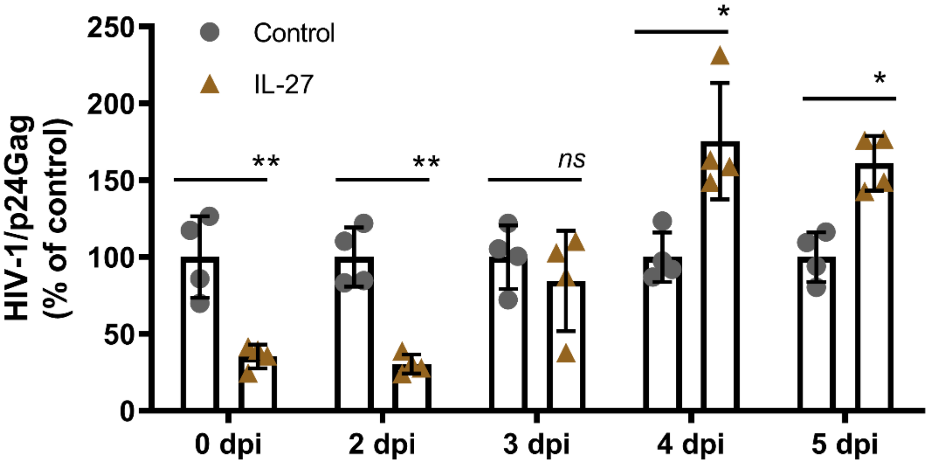

Figure S3: Time course assay of IL-27 effect on HIV-1 replication in PBMCs cultures. PBMCs were infected with HIV-1 and treated with IL-27 (100 ng/ml) at the indicated times after infection (0 dpi). At the end of the culture period (8 dpi), supernatants were collected, and viral replication was evaluated by measuring the concentration of HIV-1 p24 antigen by ELISA. *, $p<0.05$; **, $p<0.01$; Two-way ANOVA with Tukey's multiple comparisons test; n=4; error bars represent ± SD (standard deviation).

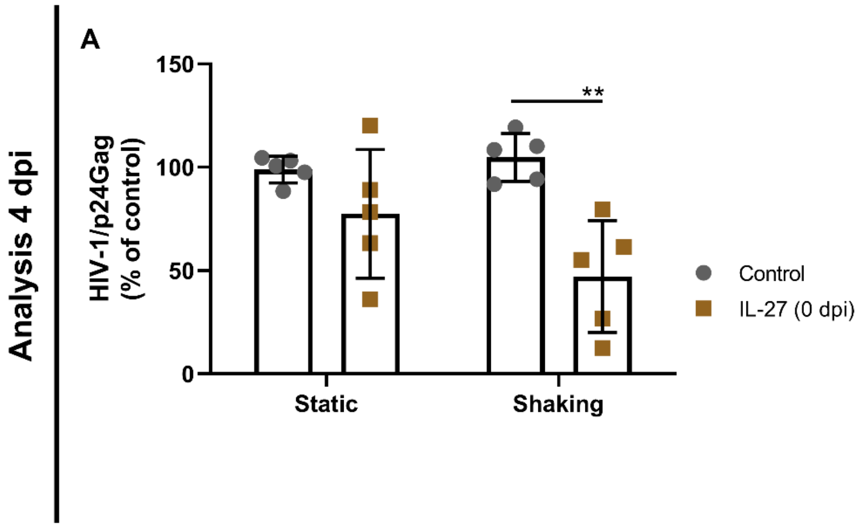

Figure S4: BST-2/Tetherin-mediated contrasting effects of IL-27 on HIV-1 infection are dependent on cell-to-cell contact. PBMCs were infected with HIV-1 and treated with IL-27 (100 ng/ml) 2 hours after infection (0 dpi). Cells were maintained under static or shaking culture conditions for 4 days. At the end of the culture period, supernatants were collected, and viral replication was evaluated by measuring the concentration of HIV-1 p24 antigen by ELISA. *, $p<0.05$; **, $p<0.01$; ***, $p<0.001$; Two-way ANOVA with Tukey's multiple comparisons test; n=5; error bars represent ± SD (standard deviation).

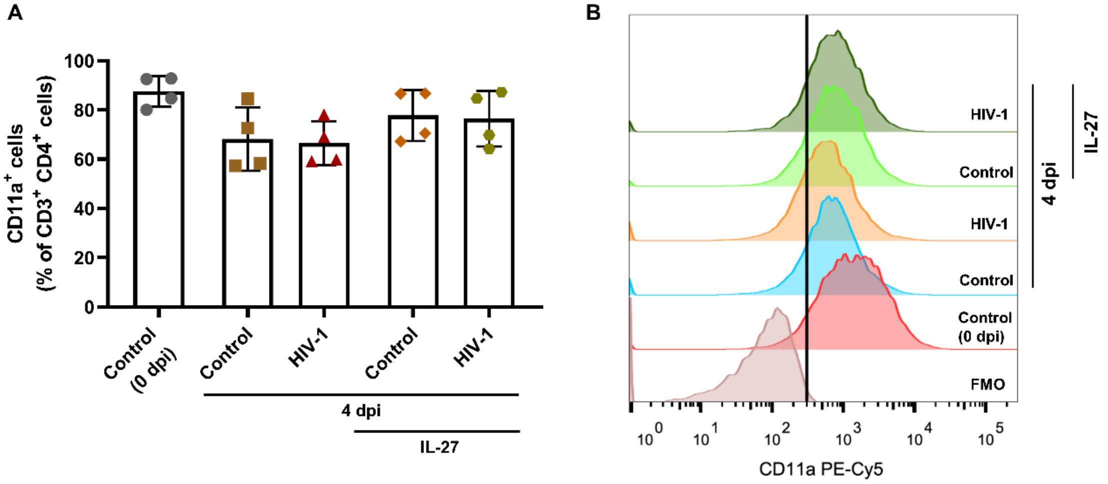

Figure S5. IL-27 does not induce the expression of CD11a. (A, B) PBMCs infected or not (Control) with HIV-1 were treated with IL-27 (100 ng/mL) two hours after infection. After 4 days of infection, the expression of CD11a was analyzed by flow cytometry within the CD3+CD4+ gate. Control (0 dpi) corresponds to uninfected cells analyzed immediately before infection and treatment. (B) Representative histogram of CD11a expression in CD3+CD4+ cells. n=4 (A and B); error bars represent ± SD (standard deviation).

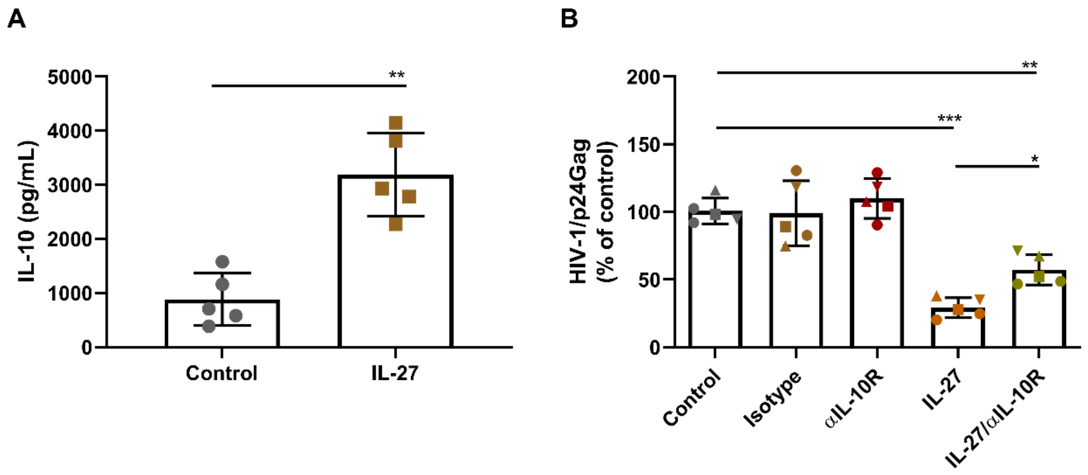

Figure S6. Participation of IL-10 in the inhibition of HIV-1 replication by IL-27. (A) PBMCs were exposed to IL-27 (100 ng/ml) and after 48 hours IL-10 levels were measured in supernatants by ELISA. (B) HIV-1-infected PBMCs were treated with IL-27 (100 ng/ml) 2 hours after infection in the presence or not of an IL-10R neutralizing antibody. Viral replication was quantified 8 days later by measuring the concentration of HIV-1 p24 antigen in the cell culture supernatants by ELISA. * $p<0.05$; **, $p<0.01$; ***, $p<0.001$; One-way ANOVA with Tukey's multiple comparisons test, n=5; error bars represent ± SD (standard deviation).

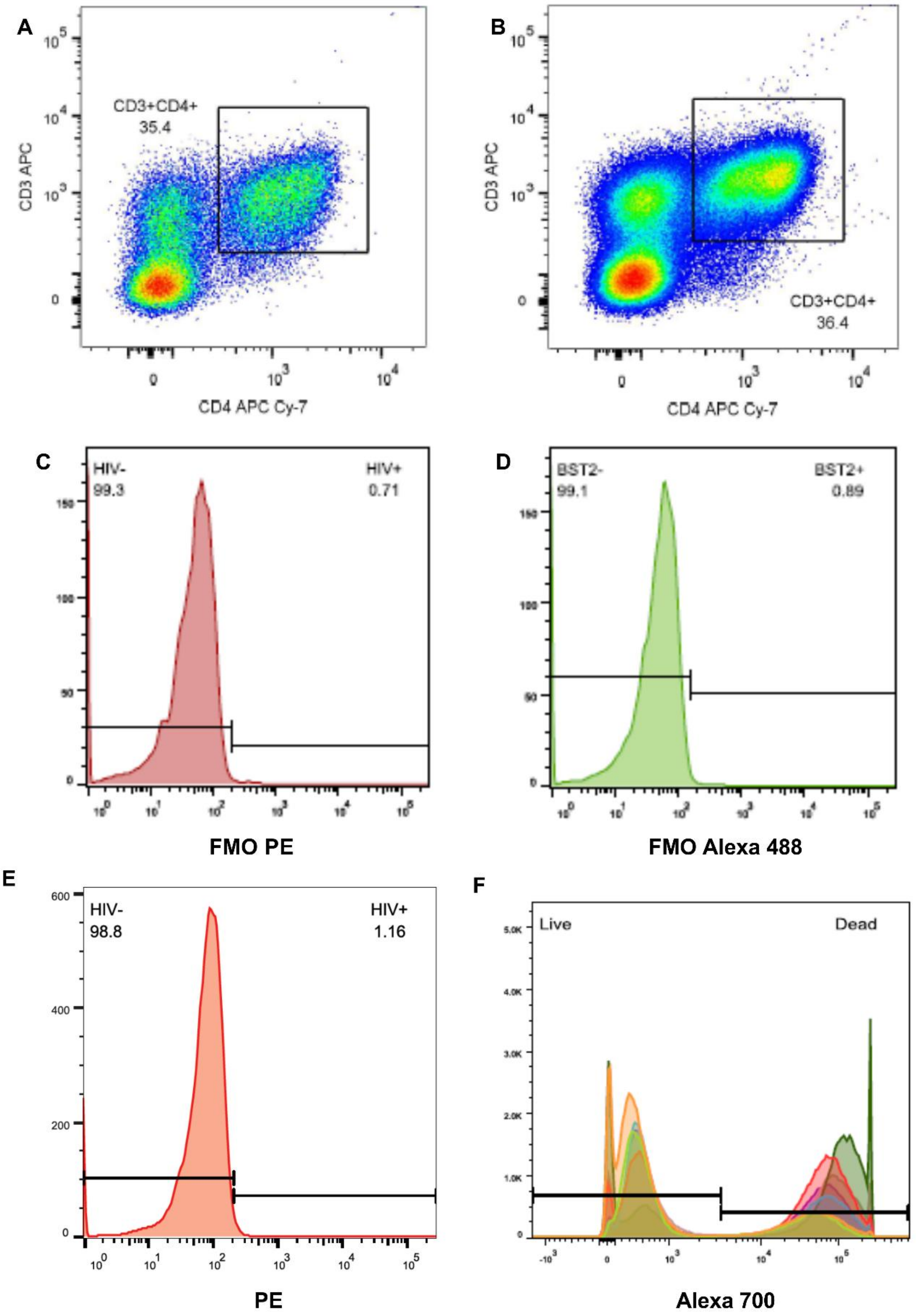
A
CD3+CD4+
35.4
CD3 APC
CD4 APC Cy-7
B
CD3+CD4+
36.4
CD3 APC
CD4 APC Cy-7
C
HIV-
99.3
HIV+
0.71
FMO PE
D
BST2-
99.1
BST2+
0.89
FMO Alexa 488
E
HIV-
98.8
HIV+
1.16
PE
F
Live
Dead
Alexa 700

Figure S7: (A, B) Representative expression of CD3 and CD4 in control (A) and IL-27-treated (B) cells. (C, D) Representative fluorescence minus one control (FMO) for HIV-1 (C) and BST-2/Tetherin (D) expression. (E) Representative control for HIV-1 antibody staining using non-infected cells. (F) Representative live/dead exclusion used for the analysis of cells in flow cytometry assays.

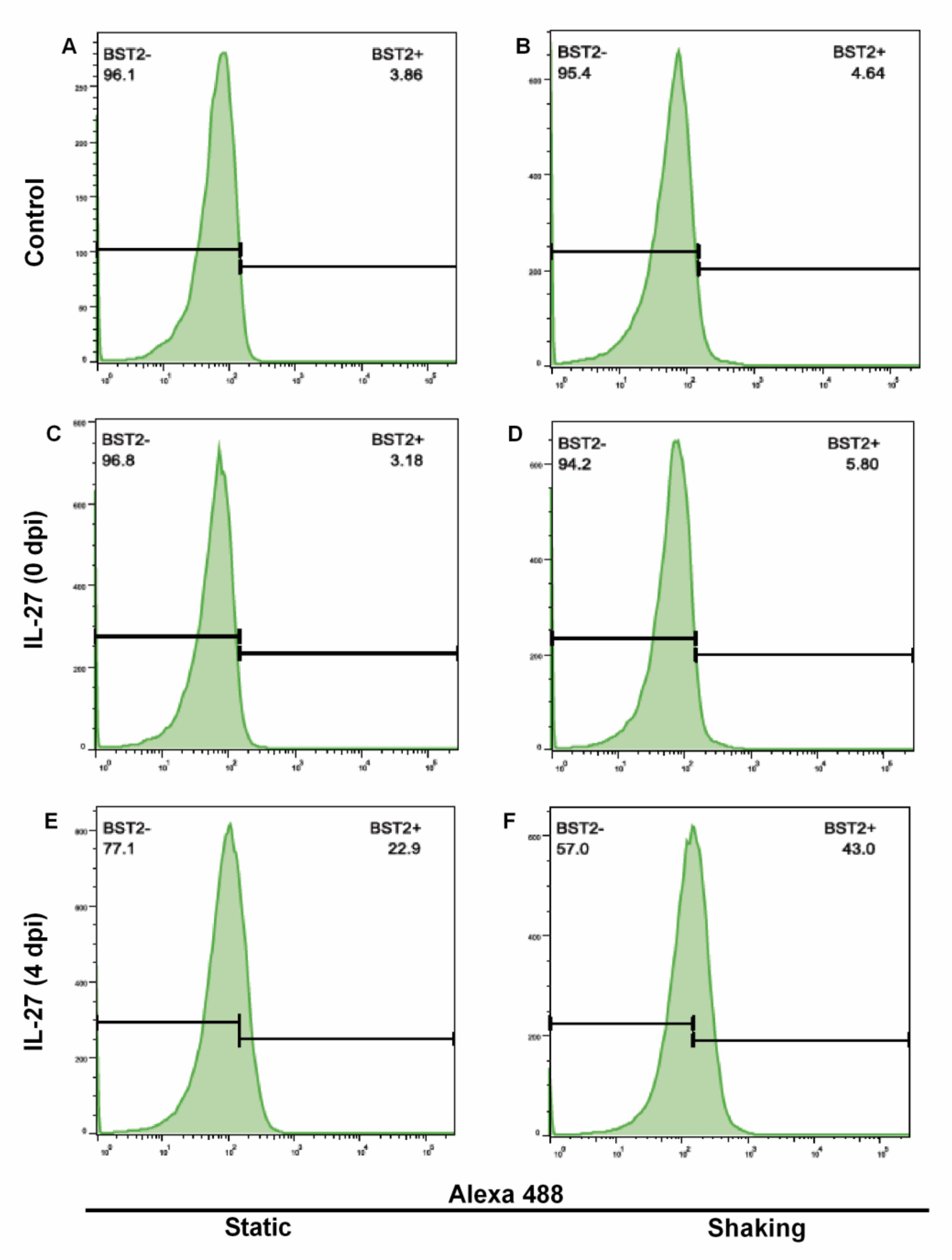

Figure S8: (A-E) Representative expression of BST-2/Tetherin in cells under static or shaking conditions exposed to IL-27 at different times post-infection. HIV-1-infected PBMCs were treated or not with 100 ng/ml of IL-27 at 0 dpi or 4 dpi and maintained at static (left panels) or shaking (right panels) conditions. (A, B) Control cells not exposed do IL-27. (C, D) Cells exposed to IL-27 at 0 dpi. (E, F) Cells exposed to IL-27 at 4 dpi.

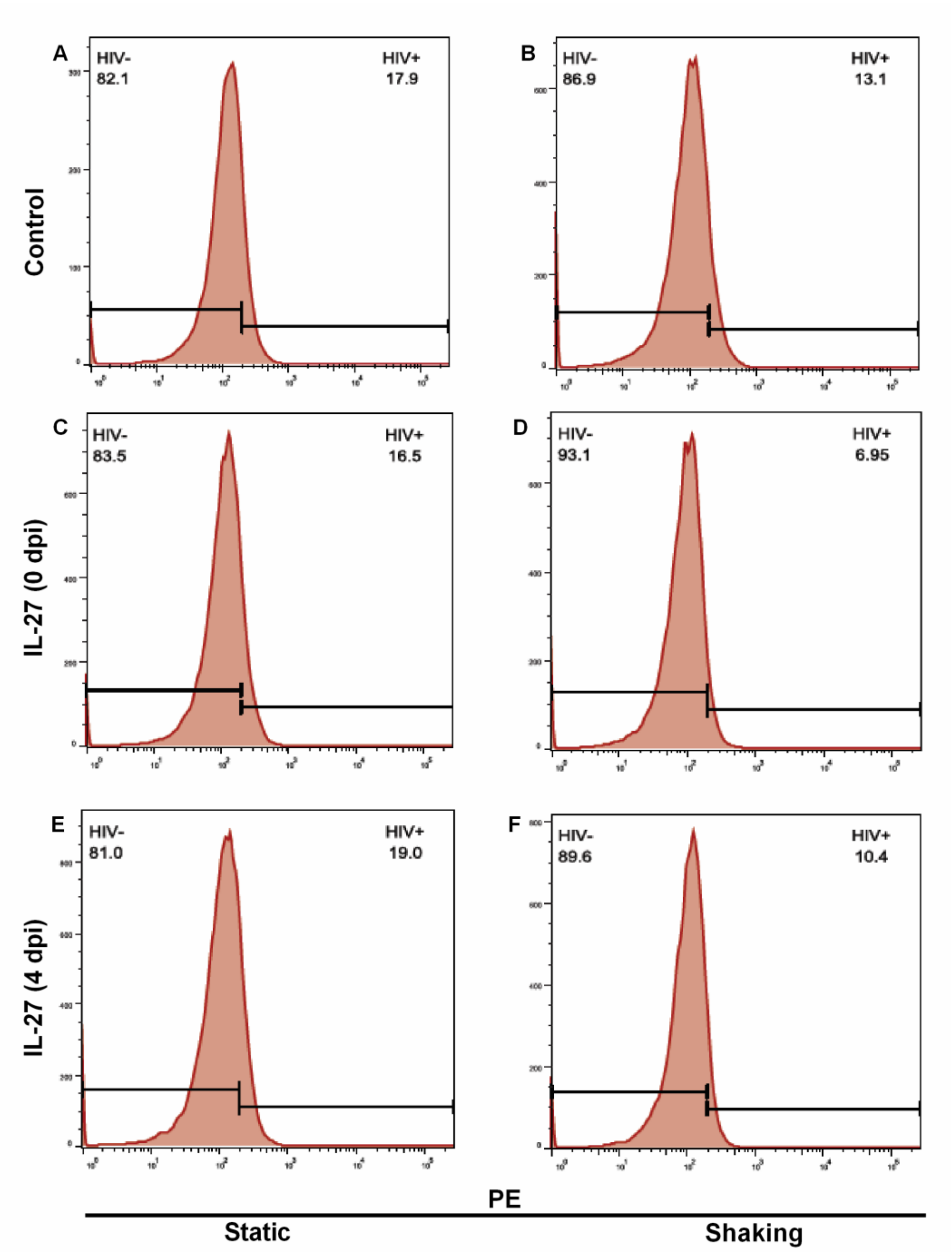

Figure S9: (A-E) Representative expression of HIV-1 in cells under static or shaking conditions exposed to IL-27 at different times post-infection. HIV-1-infected PBMCs (CCR5-tropic isolate Ba-L) were treated or not with 100 ng/ml of IL-27 at 0 dpi or 4 dpi and maintained at static (left panels) or shaking (right panels) conditions. (A, B) Control cells not exposed do IL-27. (C, D) Cells exposed to IL-27 at 0 dpi. (E, F) Cells exposed to IL-27 at 4 dpi.
